# A Bayesian model integration for mutation calling through data partitioning

**DOI:** 10.1093/bioinformatics/btz233

**Published:** 2019-03-29

**Authors:** Takuya Moriyama, Seiya Imoto, Shuto Hayashi, Yuichi Shiraishi, Satoru Miyano, Rui Yamaguchi

**Affiliations:** 1 Human Genome Center, The Institute of Medical Science, The University of Tokyo, Tokyo, Japan; 2 Health Intelligence Center, The Institute of Medical Science, The University of Tokyo, Tokyo, Japan; 3 Center for Cancer Genomics and Advanced Therapeutics, National Cancer Center, Tokyo, Japan

## Abstract

**Motivation:**

Detection of somatic mutations from tumor and matched normal sequencing data has become among the most important analysis methods in cancer research. Some existing mutation callers have focused on additional information, e.g. heterozygous single-nucleotide polymorphisms (SNPs) nearby mutation candidates or overlapping paired-end read information. However, existing methods cannot take multiple information sources into account simultaneously. Existing Bayesian hierarchical model-based methods construct two generative models, the tumor model and error model, and limited information sources have been modeled.

**Results:**

We proposed a Bayesian model integration framework named as partitioning-based model integration. In this framework, through introducing partitions for paired-end reads based on given information sources, we integrate existing generative models and utilize multiple information sources. Based on that, we constructed a novel Bayesian hierarchical model-based method named as OHVarfinDer. In both the tumor model and error model, we introduced partitions for a set of paired-end reads that cover a mutation candidate position, and applied a different generative model for each category of paired-end reads. We demonstrated that our method can utilize both heterozygous SNP information and overlapping paired-end read information effectively in simulation datasets and real datasets.

**Availability and implementation:**

https://github.com/takumorizo/OHVarfinDer.

**Supplementary information:**

Supplementary data are available at *Bioinformatics* online.

## 1 Introduction

Cancer is driven by genomic alterations. Acquired somatic mutations, together with individual germ line variations, are important factors in cancer evolution. Together with decreasing massively parallel sequencing costs, mutation calling from tumor and matched normal sequence data has become fundamental analysis methods in cancer research (Meyerson *et al.*, 2010).

Previous statistical mutation callers can be mainly categorized into two types. The first type of mutation caller does not assume any probability distribution that is specific for sequence data (Koboldt *et al.*, 2012; Yoshida *et al.*, 2011), and mutation calling is conducted based on Fisher’s exact test (Fisher, 1925). For this type, the numbers of reference supporting reads and variant supporting reads are counted in tumor and normal samples, and a *P*-value is computed based on a 2 × 2 contingency table. These methods only consider differences in variant allele frequencies between tumor and normal samples and ignore the biases found in sequence errors and mapping errors.

The second type of mutation caller constructs generative models that are specific for sequence data. This type of method first prepares generative models for sequence data, and then computes statistical scores based on techniques, e.g. maximum a posteriori inference of genotypes (Roth *et al.*, 2012) and Bayes factor-based model selection (Cibulskis *et al.*, 2013; Moriyama *et al.*, 2017; Usuyama *et al.*, 2014). The most important advantage of the second approach is that we can construct generative models based on sequence data-specific information sources, and then utilize the given information sources. Some methods are known to perform well by using some characteristic information of heterozygous single-nucleotide polymorphisms (SNPs) nearby mutation candidates (Usuyama *et al.*, 2014) or overlapping paired-end reads (Moriyama *et al.*, 2017).

Simultaneous usage of multiple characteristic information sources, e.g. heterozygous SNPs nearby mutation candidates and overlapping paired-end reads, is preferable for improving performance for the second type of method. However, existing mutation callers do not consider various information sources simultaneously. To utilize multiple information sources, we proposed a Bayesian model integration framework, named as partitioning-based model integration, and then we developed a novel mutation calling method named as OHVarfinDer based on the framework.

In Section 2, we explain the partitioning-based model integration framework, and then describe details of OHVarfinDer.

In Section 3, we first show that our method can utilize both heterozygous SNPs information and overlapping paired-end read information effectively in simulation datasets. In this experiment, we demonstrate the comparable performance of our method with other methods when only one of the two information sources is available; we also demonstrate the superior performance of our method compared to the other methods when both information sources are available. Second, we demonstrate the better performance of our method for real datasets.

In Section 4, we discuss the advantages and limitations of the proposed method.

## 2 Materials and methods

In this section, we first explain multiple characteristic information sources for mutation calling. Second, we elucidate our proposed framework of partitioning-based model integration in a general form. Third, we describe how these multiple information sources are incorporated in OHVarfinDer based on the partitioning-based model integration.

### 2.1 Characteristic information sources for mutation calling

#### 2.1.1 Heterozygous SNPs covered by paired-end reads

**Fig. 1.**
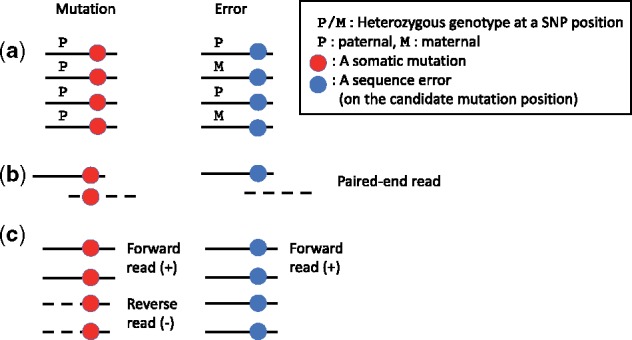
(a) The typical pattern of reads when heterozygous SNPs near the mutation candidate appear. (b) The typical pattern of paired-end reads when overlapping paired-end reads cover the mutation candidate. (c) The typical pattern of reads when both strand bias of variant supporting reads appear

The first additional information source in somatic mutation calling is heterozygous SNPs near somatic mutation candidates. The human genome is a diploid set of haplotypes, i.e. the maternal haplotype and paternal haplotype. Each somatic mutation is known to occur typically only on one side of the haplotypes, i.e. heterozygous mutation. Therefore, variant supporting reads that cover heterozygous SNPs are generated from only one side of the haplotypes as shown in the left side of Figure 1a. However, when sequence errors occur on the mutation candidate position, variant supporting reads covering heterozygous SNPs probably have both heterozygous SNPs as in the right side of Figure 1a. This information source was used in HapMuC (Usuyama *et al.*, 2014).

#### 2.1.2 Overlaps of paired-end reads

The second additional information source is overlaps of paired-end reads. Through Illumina’s sequencing, a pair of paired-end reads, i.e. forward and reverse reads, is sequenced from both sides of the same DNA fragment. If the DNA fragment is shorter than 2-fold the read length, the pair of reads has an overlapping region where sequence process is conducted twice from different directions independently.

If the both forward and reverse reads show the same alteration in the overlapping region as in the left side of Figure 1b, it is likely that the change is because of a mutation and not because of errors, as the occurrence probability of two errors at the same site in the overlapping region is expected to be very low, except for PCR errors in the sample preparation phase (Chen-Harris *et al.*, 2013). In contrast, an error case is probable when only one of the reads contains an alteration in the overlapping region as in the right side of Figure 1b. This information source has been used in OVarCall (Moriyama *et al.*, 2017).

#### 2.1.3 Strand biases of paired-end reads

The third additional information source we considered is strand biases in variant supporting reads that cover a mutation candidate. If only forward (or reverse) reads contain a mutation candidate despite sufficient numbers of both forward and reverse reads, this phenomenon is known as strand bias as in the right side of Figure 1c. If a true somatic mutation exists, strand bias rarely occurs, and the proportion of variant supporting forward/reverse reads should be ideally similar as in the left side of Figure 1c. This information source is used for filtering in MuTect (Cibulskis *et al.*, 2013).

#### 2.1.4 Representative examples in real datasets

**Fig. 2.**
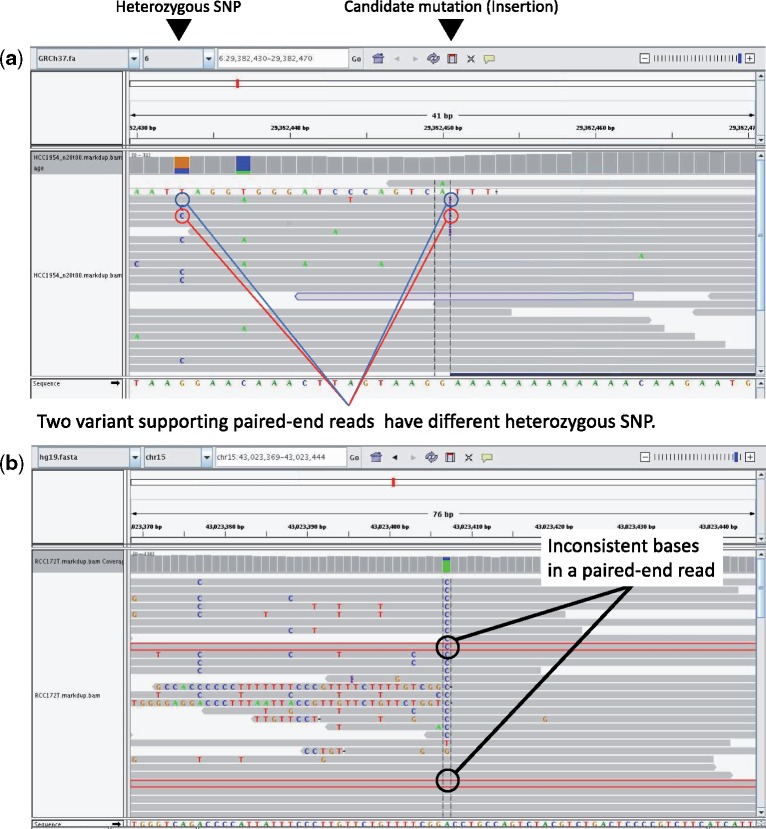
Typical cases of error are shown in the IGV screenshot. (a) In this case, both heterozygous SNPs near the mutation candidate appear in the variant supporting reads. See the erroneous case in Figure 1a. (b) One corresponding paired-end read is highlighted in red line. In this case, inconsistent bases in a paired-end read occur at a mutation candidate position. See the erroneous case in Figure 1b. Our method successfully evaluates these errors with low Bayes factor scores, i.e. 0.000059 in (a) and 0.0000011 in (b)

We show the examples from real datasets, in which we can find that given mutation candidates are only errors. Figure 2 shows screenshots of IGV (http://software.broadinstitute.org/software/igv/).

The first erroneous case shown in Figure 2a represents the variant supporting reads with both heterozygous SNPs. In this case, variant supporting reads have both heterozygous SNPs, as indicated by red and blue circles. This case corresponds with the erroneous case in Figure 1a.

The second erroneous case shown in Figure 2b represents a paired-end reads with inconsistent bases at a mutation candidate position. In this case, reads in a paired-end reads that are highlighted in red line have different bases at the mutation candidate position. This case corresponds with the erroneous case in Figure 1b.

Simpler methods, e.g. a Fisher’s exact test-based method of VarScan2, evaluate these two types of errors as somatic mutations. In the case of Figure 2a, VarScan2 showed a low *P*-value of 0.043 and in Figure 2a, VarScan2 also showed a low *P*-value of 0.0050. The main purpose of this paper is to construct a Bayesian method which discriminates these errors from somatic mutations.

### 2.2 Bayes factor for finding mutations

We denote a dataset as $X:={\left\{x_{n}\right\}}_{n=1}^{d}$, where *x_n_* is the *n*-th string consisting of {A,T,G,C} and *d* is the depth on the mutation candidate position. We denote tumor and error models as $M_{T},M_{E}$, and corresponding parameters as $\theta_{T}{,\theta }_{E}$. Next, the Bayes factor (Kass and Raftery, 1995) is written as follows:

$$\text{BF}=\frac{\mathrm{Pr}(X|M_{T})}{\mathrm{Pr}(X|M_{E})},$$

where $\mathrm{Pr}(X|M_{S})=\int Pr(X,\theta_{S}|M_{S})\mathrm{Pr}(\theta_{S}){d\theta }_{S},\;\;S\in \{T,E\}$.

### 2.3 Partitioning-based model integration

First, we assume K∈N models in each tumor and error model, and denote these models as $M_{T,k},M_{E,k}$, where k∈{1,…,K}. We denote corresponding parameters as $\theta_{T,k},\theta_{E,k}$, where k∈{1,…,K}. We also assume that we can observe indicator variable $t_{n}\in \{1,2,\ldots ,K\}$ with each data *x_n_*. We assume that the original dataset is partitioned into *K* subsets and *t_n_* indicates the subset of data to which *x_n_* belongs. We also assume that the *k*-th subset of data is generated through the *k*-th model of $M_{T,k}$ or $M_{E,k}$. We denote this augmented dataset as $X_{\text{aug}}:={\left\{(x_{n},t_{n})\right\}}_{n=1}^{d}$.

**Fig. 3.**
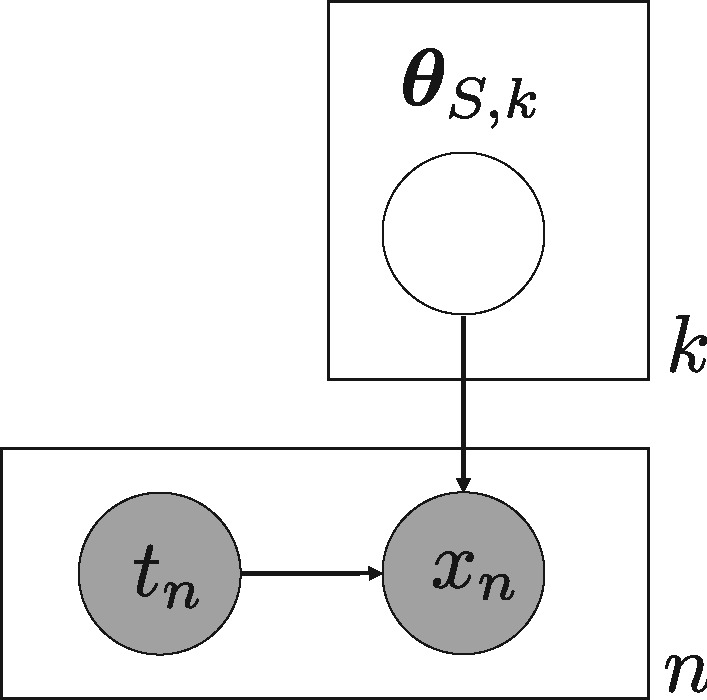
Graphical model for partitioning-based integration of generative models. Where S∈{T, E} states the hypothesis

We assume the graphical model of Figure 3 and that the distribution of each parameter $\theta_{S,k}$ is dependent on the *k*-th model of $M_{S,k}$.

(1) $$x_{n}|t_{n},\theta_{S,\text{all}},M_{S}∼\mathrm{Pr}(x_{n}|\theta_{S,t_{n}},M_{S,t_{n}}),$$

(2) $$\theta_{S,k}|M_{S}∼\mathrm{Pr}(\theta_{S,k}|M_{S,k}).$$

Our purpose here is to compute the following Bayes factor:

$$\text{BF}=\frac{\mathrm{Pr}(X_{\text{aug}}|M_{T})}{\mathrm{Pr}(X_{\text{aug}}|M_{E})}.$$

From the graphical model in Figure 3 and above assumptions of

Equations (1) and (2), the joint probability can be computed as follows:

$$\begin{array}{c} Pr(X_{\text{aug}},\theta_{S,\text{all}}|M_{S})\\ =\mathrm{Pr}(X_{\text{aug}}|\theta_{S,\text{all}},M_{S})\mathrm{Pr}(\theta_{S,\text{all}}|M_{S})\\ =\prod_{n}Pr(x_{n},t_{n}|\theta_{S,\text{all}},M_{S})\cdot \prod_{k}Pr(\theta_{S,k}|M_{S,k})\\ =\prod_{n}Pr(x_{n}|t_{n},\theta_{S,\text{all}},M_{S})\mathrm{Pr}(t_{n}|M_{S})\cdot \prod_{k}Pr(\theta_{S,k}|M_{S,k})\\ =\prod_{n}Pr(x_{n}|\theta_{S,t_{n}},M_{S,t_{n}})\mathrm{Pr}(t_{n}|M_{S})\cdot \prod_{k}Pr(\theta_{S,k}|M_{S,k}), \end{array}$$

where $S\in \{T,E\},\mathrm{Pr}(t_{n}|M_{S})>0,\theta_{S,\text{all}}:=\{\theta_{S,1},..,\theta_{S,K}\}$.

From this joint probability, the marginal likelihood can be computed as follows:

$$\mathrm{Pr}(X_{\text{aug}}|M_{S})=A_{S}\cdot \left\{\prod_{n}Pr(t_{n}|M_{S})\right\},$$

where

$$A_{S}:=\prod_{k}\int Pr(\theta_{S,k}|M_{S,k})\left\{\prod_{\left\{n|t_{n}=k\right\}}Pr(x_{n}|\theta_{S,k},M_{S,k})\right\}d\theta_{S,k}.$$

If we can assume $\mathrm{Pr}(t|M_{T})=\mathrm{Pr}(t|M_{E})$ for any t∈{1,…,K}, we do not need to set $\mathrm{Pr}(t|M_{T}),\mathrm{Pr}(t|M_{E})$ for computation of Bayes factor, because

$$\text{BF}=\frac{\mathrm{Pr}(X_{\text{aug}}|M_{T})}{\mathrm{Pr}(X_{\text{aug}}|M_{E})}=\frac{A_{T}\cdot \prod_{n}P(t_{n}|M_{T})}{A_{E}\cdot \prod_{n}P(t_{n}|M_{E})}=\frac{A_{T}}{A_{E}}.$$

This manner of model integration requires two conditions. The first condition is a partition rule on the dataset and we can construct a corresponding generative model for each partitioned dataset. The second condition is that partition probabilities should be the same among the tumor and error model ($\mathrm{Pr}(t|M_{T})=\mathrm{Pr}(t|M_{E})$). The merit of this manner is that partition probabilities $\mathrm{Pr}(t|M_{T}),\mathrm{Pr}(t|M_{E})$ do not affect the Bayes factor and thus careful and explicit settings of these probabilities are not necessary.

### 2.4 Notations in practical models of OHVarfinDer

**Fig. 4.**
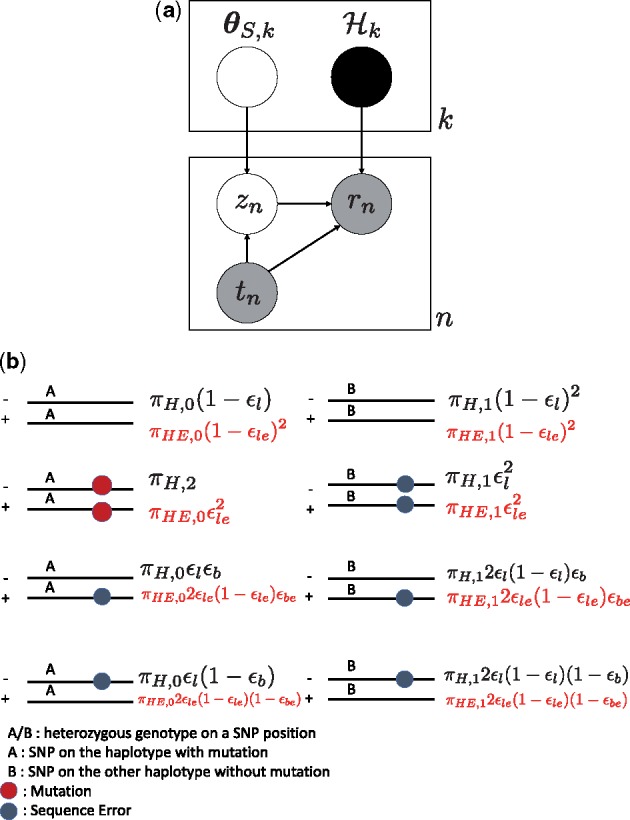
(a) Graphical model of OHVarfinDer. (b) Ideal paired-end reads set in $H_{3}$ and corresponding proportion $f_{T,3}$ and $f_{E,3}$ for the tumor model and error model. $\epsilon_{\mathrm{le}},\epsilon_{\mathrm{be}}$ and $\pi_{\mathrm{HE}}$ are error rate for overlapping reads, strand bias rate and haplotype frequency used in the error model of O(+)H(+). Characteristic information of heterozygous SNPs, overlapping paired-end reads and strand bias can be considered by setting the proportions $f_{T,3}$ and $f_{E,3}$. Occurrence probabilities shown in black (red) letters are for $f_{T,3}$ ($f_{E,3}$). The black colored formulations in the left hand side are based on the tumor model of O(+)H(−) category. The black colored formulations in the right hand side and all the red colored formulations are based on the error model of O(+)H(−), cf. the Supplementary Material A.2 and A.4

The graphical model of OHVarfinder is shown in Figure 4a. *r_n_* is the *n*-th paired-end read, i.e. tuple of two forward/reverse reads of $\left(r_{n,+},r_{n,-}\right)$, and each $r_{n,+},r_{n,-}$ is a string sequence of {A,T,G,C}. *t_n_* is the *n*-th partition indicator variable. $H_{k}$ is an set of template paired-end reads and contains paired-end reads like Figure 1. *z_n_* (if $t_{n}=k,\;z_{n}\in \{0,1,\ldots ,|H_{k}|-1\}$) is the *n*-th categorical latent variable indicating the template paired-end read for the *n*-th paired-end read *r_n_*. For the generation process of *r_n_* as a whole, we assume that *n*-th paired-end read *r_n_* is generated from *z_n_*-th paired-end read of $H_{k,z_{n}}$ with sequence errors and mapping errors added randomly.

### 2.5 Partition rules for each paired-end read in OHVarfinDer

In our method, we split paired-end reads into five types. $t_{n}\in \{0,1,2,3,4\}$ is determined for each paired-end read $r_{n,\pm }$ by the following partitioning rule.

#### 2.5.1 O(+)H(−) category

A paired-end read in this category (*t_n_* = 0) is *overlapping* between the forward read and reverse read at the mutation candidate position and *covers no* heterozygous SNPs nearby the candidate position.

#### 2.5.2 O(−)H(+) category

A paired-end read in this category (*t_n_* = 1) is *not overlapping* between the forward read and reverse read at the mutation candidate position and *covers* heterozygous SNPs nearby the candidate position.

Note that global haplotype phasing is not necessary and we only conduct haplotype phasing locally around the mutation candidate positions as previously conducted in (Usuyama *et al.*, 2014). The genotype A and B as in Figure 4b is determined from the number of variant supporting reads for each SNP.

#### 2.5.3 O(+)H(+) category

A paired-end read in this category (*t_n_* = 2) is *overlapping* between the forward read and reverse read at the mutation candidate position and *covers* heterozygous SNPs nearby the candidate position.

#### 2.5.4 O(−)H(−)S(+) category

A paired-end read in this category (*t_n_* = 3) is *not overlapping* between the forward read and reverse read at the mutation candidate position and *covers no* heterozygous SNPs nearby the candidate position. The mutation candidate position is covered by the *forward* read. (Forward/reverse is determined by the mapping direction compared to the reference sequence.)

#### 2.5.5 O(−)H(−)S(−) category

A paired-end read in this category (*t_n_* = 4) is *not overlapping* between the forward read and reverse read at the mutation candidate position and *covers no* heterozygous SNPs nearby the candidate position. The mutation candidate position is covered by the *reverse* read.

#### 2.5.6 Suitability of partition based integration

We should note that partitioning-based integration is suited for this problem setting for two reasons. The first reason is that we can set partition rules on paired-end reads and construct generative models for each dataset by referring to existing methods. The second reason is that partitioning probabilities $\mathrm{Pr}(t|M_{T}),\mathrm{Pr}(t|M_{E})$ are thought to be the same, e.g. the existence of a mutation does not affect whether a paired-end read will cover a heterozygous SNP.

### 2.6 Details of tumor generative model for O(+)H(+) type of paired-end read

Here, we only show the details of the tumor generative model for O(+)H(+) type (*t_n_* = 3) due to the limitation of the space. See the Supplementary Material A.1–A.8 for the details of our models. *z_n_* is the one of eight expression vector indicating an idealized paired-end read. For the parameters, we used $\epsilon_{l},\epsilon_{b},\pi_{H}$ (that is $\theta_{T,3}:=(\epsilon_{l},\epsilon_{b},\pi_{H})$). $\epsilon_{l}\in [0,1]$ is the error rate when the paired-end read is overlapping at the mutation candidate position, $\epsilon_{b}\in [0,1]$ is the strand bias rate. $\pi_{H}$ is a 3-dimensional non-negative simplex, indicating the proportion of paired-end reads from a maternal haplotype, paternal haplotype and haplotype with somatic mutation. Let $\alpha_{l}\in R_{+}^{2},\alpha_{b}\in R_{+}^{2},\gamma_{H}\in R_{+}^{3}$ the hyperparameters for $\epsilon_{l},\epsilon_{b},\pi_{H}$. The tumor generative model for the O(+)H(+) type of paired-end read is defined as follows:

$$\begin{array}{c} \epsilon_{l}|\alpha_{l}∼P_{\text{beta}}(\epsilon_{l}|\alpha_{l}),\\ \epsilon_{b}|\alpha_{b}∼P_{\text{beta}}(\epsilon_{b}|\alpha_{b}),\\ \pi_{H}|\gamma_{H}∼P_{\text{dir}}(\pi_{H}|\gamma_{H}),\\ z_{n}|\epsilon_{l},\epsilon_{b},\pi_{H},t_{n}∼P_{\text{mult}}(z_{n}|f_{T,t_{n}}),\\ r_{n}|z_{n},H_{t_{n}},\\ ∼P_{\text{align}}(r_{n,+}|H_{t_{n},\mathrm{idx}(z_{n}),+})P_{\text{align}}(r_{n,-}|H_{t_{n},\mathrm{idx}(z_{n}),-}). \end{array}$$

where $P_{\text{beta}}(\cdot ),P_{\text{dir}}(\cdot ),P_{\text{mult}}(\cdot )$ are the probability density function of beta, Dirichlet, and multinomial distributions respectively. $P_{\text{align}}(\cdot )$ is the alignment probability which is formulated by profile hidden Markov model (HMM) (Albers *et al.*, 2011; Usuyama *et al.*, 2014). $f_{T,k}$ is a non-negative simplex defined by $\theta_{T,k}$. We only show the case for $f_{T,3},f_{E,3}$ in Figure 4b. idx(·) is a function that returns the index where the value is 1 from a given one-hot encoding vector.

### 2.7 Bayes factor in OHVarfinDer

Here, we show the Bayes factor in OHVarfinDer and explain that our method is truly based on the partitioning-based integration and that setting of $\mathrm{Pr}(t|M_{T})$ and $\mathrm{Pr}(t|M_{E})$ are not necessary. Let $R_{\text{NT}}:={\left\{r_{n}\right\}}_{n=1}^{d}$ is the set of paired-end reads for both tumor and normal sample data which cover a mutation candidate position, and the marginal likelihoods can be computed as follows:

$$\begin{array}{c} Pr(R_{\text{NT}},{\left\{t_{n}\right\}}_{n}|M_{S})\\ =\left\{\prod_{n}Pr(t_{n}|M_{S})\right\}\cdot \prod_{k=0}^{4}\int F_{k}d\theta_{S,k}\prod_{\left\{n|t_{n}=k\right\}}dz_{n}, \end{array}$$

where

$$\begin{array}{c} F_{k}=\mathrm{Pr}(\theta_{S,k}|M_{S,k})\prod_{\left\{n|t_{n}=k\right\}}Pr(z_{n}|\theta_{S,k})\mathrm{Pr}(r_{n}|z_{n},H_{t_{n}})\\ (=\mathrm{Pr}(R_{\text{NT}}|M_{S},{\left\{t_{n}\right\}}_{n}). \end{array}$$

Therefore, if $\mathrm{Pr}(t|M_{T})=\mathrm{Pr}(t|M_{E})$, it is not necessary to set these distributions in the Bayes factor of OHVarfinDer as shown in the previous section.

### 2.8 Computation of marginal likelihoods

We applied the variational Bayes procedures for computing $\mathrm{Pr}(R_{\text{NT}}|M_{S})$. We can obtain a lower bound for $\ln\mathrm{Pr}(R_{\text{NT}}|M_{S},{\left\{t_{n}\right\}}_{n})$ from the convexity of *log* function (Jensen, 1906).

(3) $$\ln\mathrm{Pr}(R_{\text{NT}}|M_{S},{\left\{t_{n}\right\}}_{n})\geq E_{q}\left[\ln\frac{\mathrm{Pr}(R_{\text{NT}},Z_{S,\text{NT}}|M_{S},{\left\{t_{n}\right\}}_{n})}{q(Z_{S,\text{NT}})}\right],$$

where we denote all latent variables and parameters of ${\left\{z_{n}\right\}}_{n},{\left\{\theta_{S,k}\right\}}_{k}$ as $Z_{S,\text{NT}}$. $q(Z_{S,\text{NT}})$ is the variational distribution for $Z_{S,\text{NT}}$ which is formulated in the independent form as follows:

$$q(Z_{S,\text{NT}}):=\prod_{k}\left[q(Z_{S,\text{NT},k})q(\theta_{S,k})\right],q(Z_{S,\text{NT},k}):=\prod_{n|t_{n}=k}q(z_{n}).$$

In the above inequality of Equation (3), the equality holds true when $q(Z_{S,\text{NT}})$ is equal to the posterior distribution of the $\mathrm{Pr}(Z_{S,\text{NT}}|R_{\text{NT}},{\left\{t_{n}\right\}}_{n},M_{S})$. In the variational Bayes procedure (Beal, 2003), we maximize the lower bound for each variational distribution of $q(\theta_{S,k})$ and $q(Z_{S,\text{NT},k})$ iteratively until the updated lower bound converges, and approximate the log marginal likelihood using this maximized lower bound. We described the full procedures for variational Bayes in the Supplementary Material A.9–A.16.

## 3 Results

### 3.1 Performance evaluation of OHVarfinDer using simulation data

#### 3.1.1 Simulation data generation procedure

We tested OHVarfinDer using simulation datasets. The simulation procedure is described as follows. In the following procedure, we prepared two types of errors. The first type of errors are position-specific ones, and known as error prone sites (Moriyama *et al.*, 2017; Shiraishi *et al.*, 2013). The second type of errors are non-position-specific ones.

1. Generate a random reference DNA sequence.
2. Generate a heterozygous germ line variant in a random location, as well as two haplotypes (h1 and h2)
3. Generate a somatic mutation randomly around a heterozygous germ line variant, according to an empirical distribution of whole genome data, as well as two haplotypes (h3 and h4)
4. Randomly generate paired-end reads around 900 somatic mutations and 2100 error prone sites randomly.
  - Determine the number of paired-end reads covering the position, by generating a random value *d* from a norm distribution of *N*(50, 2), and round *d* to the nearest integer value.
  - Randomly determine the haplotype of the original DNA fragment. We set the frequency of haplotypes as h1: 50-*v*%, h2: 50%, h3: *v*%, h4: 0% if a somatic mutation truly exists. We set the frequency of haplotypes as h1: 50%, h2: 50%, h3: 0%, h4: 0% otherwise.
  - For each paired-end read, determine the DNA fragment size by generating a random value *l* from $N(\mu_{l},\sigma_{l})$, and round *l* to the nearest integer value.
  - Generate the 100-bp length read sequence on forward strand. Each observed base flips with the sequence error probability of *p*_error_. If the position of each observed base is the error prone site, *p*_error_ is generated from a beta distribution of *Beta*(2, 30). If the position of each observed base is not the error prone site, *p*_error_ is generated from *Beta*(10, 1000).
  - Generate the read sequence on the reverse strand like (d).

#### 3.1.2 Performance evaluation of OHVarfinDer using simulation data

As a counterpart method, we prepared OVarCall, HapMuC, and a simple Fisher’s exact test (Fisher, 1925) method, which uses a 2 × 2 contingency table of read counts, tumor and normal samples/variant and reference alleles. We calculated the area under the curve (AUC) values from the plotted receiver-operating characteristic curve (ROC) (Bradley, 1997) for each simulation condition as shown in Table 1. We described the filter conditions in the Supplementary Material B.1.

**Table 1.** Simulation results summary (AUC)

	*v* (%)	Heterozygous SNPs	Overlap	Distance to SNP	μ_*l*_	σ_*l*_	OHVarfinDer	OVarCall	HapMuC	Fisher	#SNV	#Error
**A**	5	−	−	500–5000	300	30	*0.828*	0.750	*0.828*	0.810	341	822
	10	−	−				*0.891*	0.867	0.880	*0.891*	713	871
	20	−	−				0.967	0.978	0.950	*0.983*	896	872
**B**	5	−	+	500–5000	180	30	*0.938*	0.917	0.786	0.817	407	1394
	10	−	+				*0.958*	0.954	0.843	0.899	763	1413
	20	−	+				0.989	*0.991*	0.947	0.988	897	1411
**C**	5	+	−	1–100	300	30	0.880	0.765	*0.882*	0.825	301	851
	10	+	−				*0.916*	0.877	0.907	0.886	733	871
	20	+	−				*0.986*	0.984	0.977	0.983	896	925
**D**	5	+	+	1–100	180	30	*0.943*	0.923	0.838	0.803	388	1356
	10	+	+				*0.975*	0.952	0.918	0.914	757	1398
	20	+	+				*0.994*	0.991	0.977	0.990	896	1354

The highest AUC values are written in italic letters.

In the simulation dataset under the condition of **B**, only overlapping paired-end read information was available. In this case, our method performs comparable with OVarCall. In the simulation dataset in the condition of **C**, only heterozygous SNP information was available. In this case, our method performed comparably well with HapMuC that can utilize this information source. In the simulation dataset under the condition of **A**, neither of the above types of information was available. In this case, our method performed comparably well with Fisher’s exact test. In the simulation dataset under the condition of **D**, both overlapping paired-end read information and heterozygous SNP information were available. In this case, our method outperformed both OVarCall and HapMuC. We summarized the ROC curves in the Supplementary Material B.10.1–B.10.3.

### 3.2 Performance evaluation of OHVarfinDer using real data

#### 3.2.1 SNVs in exome sequence dataset

We confirmed whether the performance of our method could be improved by using overlapping information using real exome datasets, as shown in

Table 2 for the real datasets, we used exome sequence data from renal clear-cell carcinoma, which has already been used for performance evaluation of OVarCall (Moriyama *et al.*, 2017). In these datasets, ∼40% of paired-end reads overlapped, and thus the use of overlapping paired-end reads is expected to affect the performance. In this dataset, true somatic SNVs were validated by deep sequencing (Shiraishi *et al.*, 2013). In both the case of lower variant allele frequency of 2–7% and the case of moderate variant allele frequency above 7%, OHVarfinDer performed comparably well with OVarCall and outperformed HapMuC. Furthermore, we observed that our method returned low Bayes factor of 0.0000011 in the false positive case in Figure 2b. Therefore, we confirmed that our method can incorporate overlapping information and improve its performance. For the details of this experiment, see the Supplementary Material B.2, B.8 and B.10.4.

**Table 2.** Exome datasets summary (AUC)

SNV/InDel	VAF	OVarCall	OHVarfinDer	HapMuC	Strelka	MuTect	VarScan2	#SNV	#Error
SNV	2–7%	0.982	*0.990*	0.965	0.933	0.875	0.625	52	2422
SNV	≥7%	0.991	0.988	0.955	*0.995*	0.994	0.900	184	1982

The highest AUC values are written in italic letters. VAF, represents variant allele frequency; SNV, represents single nucleotide variant.

#### 3.2.2 SNVs and InDels in whole genome dataset

We examined whether we could improve the performance of our method by using heterozygous SNP and strand bias information using whole genome sequence data. The results are summarized in

Table 3 for the dataset, we used whole genome sequence datasets from breast cancer cell lines, which are publicly available as a part of The Cancer Genome Atlas (TCGA) Mutation Calling Benchmark 4 datasets (These datasets can be downloaded from https://gdc.cancer.gov/resources-tcga-users/tcga-mutation-calling-benchmark-4-files) and have been used for performance evaluation of HapMuC.

**Table 3.** Real datasets summary whole genome (AUC)

Sample	SNV/InDel	OVarCall	OHVarfinDer	HapMuC	Strelka	MuTect	VarScan2	#SNV/InDel	#Error
HCC1143_n20t80	SNV	0.869	*0.906*	0.827	0.873	0.848	0.801	10 618	2327
HCC1143_n40t60		0.870	*0.901*	0.824	0.877	0.855	0.799	8517	2049
HCC1143_n60t40		0.884	*0.912*	0.843	0.901	0.876	0.814	5450	1684
HCC1143_n80t20		0.901	*0.941*	0.870	0.938	0.918	0.830	1874	1451
HCC1954_n20t80		0.882	*0.934*	0.852	0.903	0.869	0.862	10 653	2854
HCC1954_n40t60		0.893	*0.941*	0.852	0.917	0.880	0.858	7969	2327
HCC1954_n60t40		0.917	*0.949*	0.865	0.937	0.905	0.852	4638	1770
HCC1954_n80t20		0.941	0.970	0.880	*0.972*	0.942	0.848	1389	1404
Total		0.895	*0.935*	0.860	0.913	0.886	0.852	51 108	15 866
HCC1143_n20t80	InDel	0.707	*0.796*	0.678	0.713	—	0.722	926	4951
HCC1143_n40t60		0.733	*0.814*	0.700	0.755	—	0.748	617	4761
HCC1143_n60t40		0.760	*0.834*	0.723	0.784	—	0.778	328	4563
HCC1143_n80t20		0.809	*0.855*	0.770	0.816	—	0.800	94	4899
HCC1954_n20t80		0.800	*0.860*	0.771	0.822	—	0.825	1771	5219
HCC1954_n40t60		0.821	*0.866*	0.778	0.843	—	0.835	1172	5215
HCC1954_n60t40		0.819	*0.863*	0.770	0.848	—	0.831	607	5200
HCC1954_n80t20		0.815	*0.887*	0.777	0.864	—	0.823	159	5053
Total		0.777	*0.838*	0.774	0.794	—	0.792	5674	39 861

The highest AUC values are written in italic letters.

In these datasets, pure cell line sequence datasets of normal and tumor cell line and computational mixtures of these sequence datasets are prepared, e.g. HCC1143_n40t60 represents that 40% of pure normal and 60% of pure tumor sequence data are mixed. In this experiment, we obtained answers of true mutations from these pure cell line datasets, and we conducted performance evaluations for tumor sequence datasets with several mixture rates, i.e. n20t80, n40t60, n60t40, n80t20. For these datasets, the use of heterozygous SNPs information and strand bias information is important for improving performance because the average proportion of overlapping paired-end reads was ∼3% within these datasets.

For the performance of OHVarfinDer, OHVarfinDer performed better than any other mutation caller, except for HCC1954_n80t20. We also observed that our method returned low Bayes factor of 0.000059 in the false positive case in Figure 2a. Therefore, we confirmed that our method can incorporate heterozygous SNP and strand bias information and improve its performance. For the details of this experiment, see the Supplementary Material B.3, B.9 and B.10.5.

## 4 Discussion

Some mutation calling methods, e.g. HapMuC and OVarCall, can incorporate a characteristic information source, e.g. heterozygous SNPs and overlapped paired-end reads, in their mutation calling process. However, no existing methods utilize multiple types of such characteristic information sources simultaneously.

In this paper, we first introduced a framework for Bayesian model integration named as partitioning-based model integration, which differs from Bayesian model averaging (Hoeting *et al.*, 1999). In this framework, we first set a partitioning rule for data and augmented the data with indicator variables which show the category of partitioning. Second, we constructed a generative model for each category of partitioned dataset. This framework requires two assumptions. The first assumption is that we can set a partitioning rule and construct corresponding generative models. The second assumption is that partitioning probabilities are common among the tumor model and error model. If the above assumptions hold true, we can compute the Bayes factor without careful setting of prior partitioning probabilities. In our problem setting of mutation calling, the above two assumptions seem natural, and thus we constructed a Bayesian mutation calling method, OHVarfinDer, based on this framework.

We conducted performance evaluations with simulation and real datasets. In the simulation datasets, we showed that our method could utilize multiple information sources, particularly overlapping paired-end read information and heterozygous SNP information. If only one information source was given, our method performed comparably well with other existing methods. If both information sources were given, our method performed better than other existing methods. In the real datasets, e.g. TCGA Mutation Calling Benchmark 4 datasets, we also demonstrated the better performance of our method compared to other existing methods.

We have demonstrated how to integrate known multiple information sources for mutation calling by our framework. We note that mapping quality and base quality of reads are also used in our method by incorporating the profile HMM modeling (Albers *et al.*, 2011; Usuyama *et al.*, 2014). Although our framework is practically useful for mutation calling, there is at least one limitation for this framework, i.e. our framework does not assume inference over the parameter distributions, e.g. prior distributions for the error parameters. Such inference is important if we consider using multiple sequence datasets simultaneously. For example, if we can use pooled normal sequence datasets, we can infer the error distributions depending on the genomic positions. For the future work, we plan to extend our framework to infer the form of the parameter distributions, e.g. incorporating predictive distributions for the error parameters.

## Supplementary Material

btz233_Supplementay_Appendix
