# Supplementary material for: A Bayesian model integration for mutation calling through data partitioning
Source: Bioinformatics. 2019 Mar 29;35(21):4247–54. doi: 10.1093/bioinformatics/btz233 (PMC6821361; doi:10.1093/bioinformatics/btz233)
Supplement: btz233_Supplementay_Appendix [file btz233_supplementay_appendix.pdf]

Supplementary Materials

Contents

A Details of Method **3**
A.1 Notations for Generative Models in OHVarfinDer 3
A.2 O(+)H(-) Category 3
A.3 O(-)H(+) Category 4
A.4 O(+)H(+) Category 5
A.5 O(-)H(-)S(+) Category 6
A.6 O(-)H(-)S(-) Category 7
A.7 All the parameters and hyperparameters for tumor generative model in OHVarfinDer 9
A.8 Joint Probability for Tumor Generative Model 9
A.9 Assumptions on Variational Distributions 10
A.10 Lower Bound for Marginal Likelihoood in Tumor Generative Model 10
A.11 Maximize $\mathcal{L}_T(q)$ with respect to $q(\boldsymbol{\pi}_H)$ 10
A.12 Maximize $\mathcal{L}_T(q)$ with respect to $q(\boldsymbol{\pi}_F)$ 11
A.13 Maximize $\mathcal{L}_T(q)$ with respect to $q(\epsilon_l)$ 12
A.14 Maximize $\mathcal{L}_T(q)$ with respect to $q(\epsilon_h)$ 13
A.15 Maximize $\mathcal{L}_T(q)$ with respect to $q(\epsilon_b)$ 13
A.16 Maximize $\mathcal{L}_T(q)$ with respect to $q(\boldsymbol{z}_{D,n}^{(k)})$ 14
A.17 All the Parameters and Hyperparameters for Error Generative Model in OHVarfinDer 16
A.18 Different Error Generative Models for Whole and Exome Data Sets 17
A.19 Joint Probability for Error Generative Model in Whole Genoeme Sequence Data 18
A.20 Joint Probability For Error Generative Model in Exome Sequence Data 19
A.21 Prior Hyperparameters Used in Performance Evaluation . . . 20

B Details of Result **21**
B.1 Filter Conditions for Simulation Data Sets 21
B.2 Filter Conditions for Exome Sequence Data 22
B.3 Filter Conditions for Whole Genome Sequence Data 23
B.4 The Criteria for Calling Somatic Mutation on the Pure Datasets of the TCGA Mutation Calling Benchmark 4 Datasets 24
B.5 Work Flow of Mutation Calling and Performance Evaluation 25
B.6 Parameters for Alternative Methods in Exome Sequence Data 26
B.7 Parameters for Alternative Methods in Whole Genome Data . 26
B.8 Basic Information about Original Exome Sequence Data . . . 27

B.9 Basic Information about Original Whole Genome Sequence Data 28
B.10 ROC Curves in Performance Evaluation 29
B.10.1 ROC Curves for Simulation Data Sets with 5 % Variant Allele Frequency 29
B.10.2 ROC Curves for Simulation Data Sets with 10 % Variant Allele Frequency 30
B.10.3 ROC Curves for Simulation Data Sets with 20 % Variant Allele Frequency 31
B.10.4 ROC Curves for SNVs in RCC Data Sets 32
B.10.5 ROC Curves for SNVs in TCGA Benchmark Data Sets 33
B.10.6 ROC Curves for InDels in TCGA Benchmark Data Sets 34
B.11 Comparison with the Gold Standard Data Sets in ICGC . . . 35

A Details of Method

A.1 Notations for Generative Models in OHVarfinDer

In order to express the difference of tumor and normal data, we denote the n-th paired-end read from tumor sample as $r_{T,n}$, and denote the n-th normal sample paired-end read as $r_{N,n}$. (We represent the n-th partition category as $t_{T,n}, t_{N,n}$ in the same way.) As for the latent variables, in order to express not only the difference of samples but also the category of partition, we denote the n-th latent variable of k-th category in the tumor sample as $z^{(k)}_{T,n}$, and denote the n-th latent variable of k-th category in the normal sample as $z^{(k)}_{N,n}$.

A.2 O(+)H(-) Category

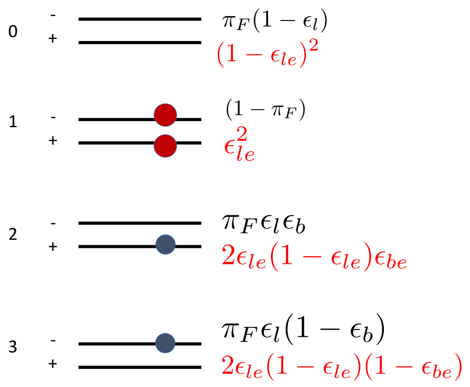

Figure 1:

Here we define functions reprepenting the logarithm of a paired-end read $r_{D,n}$ generation probability (where $t_{D,n}=0$) given latent variable and parameters, as follows.

$$
\begin{aligned}
&L_{T,O}(r_{D,n}, z^{(0)}_{D,n}, \pi_F, \epsilon_l, \epsilon_b)\\
&:= z^{(0)}_{D,n,0}\left\{\ln \pi_F(1-\epsilon_l) + \ln P(\boldsymbol{r}_{D,n}|\mathcal{H}_{idx(z^{(0)}_{D,n,0})})\right\}\\
&\quad + z^{(0)}_{D,n,1}\left\{\ln(1-\pi_F) + \ln P(r_{D,n}|\mathcal{H}_{idx(z^{(0)}_{D,n,1})})\right\}\\
&\quad + z^{(0)}_{D,n,2}\left\{\ln \pi_F\epsilon_l\epsilon_b + \ln P(r_{D,n}|\mathcal{H}_{idx(z^{(0)}_{D,n,2})})\right\}\\
&\quad + z^{(0)}_{D,n,3}\left\{\ln \pi_F\epsilon_l(1-\epsilon_b) + \ln P(r_{D,n}|\mathcal{H}_{idx(z^{(0)}_{D,n,3})})\right\}, D \in \{T, N\}
\end{aligned}
$$

$$
\begin{aligned}
&L_{E,O}(r_{D,n}, z^{(0)}_{D,n}, \epsilon_{le}, \epsilon_{be}) \\
&:= z^{(0)}_{D,n,0}\left\{2\ln(1-\epsilon_{le}) + \ln P(r_{D,n}|\mathcal{H}_{idx(z^{(0)}_{D,n,0})})\right\} \\
&\quad + z^{(0)}_{D,n,1}\left\{2\ln\epsilon_{le} + \ln P(r_{D,n}|\mathcal{H}_{idx(z^{(0)}_{D,n,1})})\right\} \\
&\quad + z^{(0)}_{D,n,2}\left\{\ln 2\epsilon_{le}(1-\epsilon_{le})\epsilon_{be} + \ln P(r_{D,n}|\mathcal{H}_{idx(z^{(0)}_{D,n,2})})\right\} \\
&\quad + z^{(0)}_{D,n,3}\left\{\ln 2\epsilon_{le}(1-\epsilon_{le})(1-\epsilon_{be}) + \ln P(r_{D,n}|\mathcal{H}_{idx(z^{(0)}_{D,n,3})})\right\}, D \in \{T, N\}
\end{aligned}
$$

A.3 O(-)H(+) Category

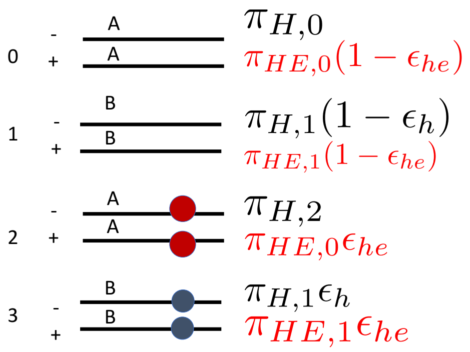

Figure 2:

Here we define functions reprepenting the logarithm of a paired-end read $r_{D,n}$ generation probability (where $t_{D,n} = 1$) given latent variable and parameters, as follows.

$$
\begin{aligned}
&L_{T,H}(r_{D,n}, z^{(1)}_{D,n}, \boldsymbol{\pi}_H, \epsilon_h) \\
&:= z^{(1)}_{D,n,0}\left\{\ln\pi_{H,0} + \ln P(r_{D,n}|\mathcal{H}_{idx(z^{(1)}_{D,n,0})})\right\} \\
&\quad + z^{(1)}_{D,n,1}\left\{\ln\pi_{H,1}(1-\epsilon_h) + \ln P(r_{D,n}|\mathcal{H}_{idx(z^{(1)}_{D,n,1})})\right\} \\
&\quad + z^{(1)}_{D,n,2}\left\{\ln\pi_{H,2} + \ln P(r_{D,n}|\mathcal{H}_{idx(z^{(1)}_{D,n,2})})\right\} \\
&\quad + z^{(1)}_{D,n,3}\left\{\ln\pi_{H,1}\epsilon_h + \ln P(r_{D,n}|\mathcal{H}_{idx(z^{(1)}_{D,n,3})})\right\}, D \in \{T, N\}
\end{aligned}
$$

$$
\begin{aligned}
&L_{E,H}(r_{D,n}, z^{(1)}_{D,n}, \pi_{HE}, \epsilon_{he}) \\
&:= z^{(1)}_{D,n,0}\left\{\ln \pi_{HE,0}(1-\epsilon_{he}) + \ln P(r_{D,n}|\mathcal{H}_{idx(z^{(1)}_{D,n,0})})\right\} \\
&\quad + z^{(1)}_{D,n,1}\left\{\ln \pi_{HE,1}(1-\epsilon_{he}) + \ln P(r_{D,n}|\mathcal{H}_{idx(z^{(1)}_{D,n,1})})\right\} \\
&\quad + z^{(1)}_{D,n,2}\left\{\ln \pi_{HE,0}\epsilon_{he} + \ln P(r_{D,n}|\mathcal{H}_{idx(z^{(1)}_{D,n,2})})\right\} \\
&\quad + z^{(1)}_{D,n,3}\left\{\ln \pi_{HE,1}\epsilon_{he} + \ln P(r_{D,n}|\mathcal{H}_{idx(z^{(1)}_{D,n,3})})\right\}, D \in \{T, N\}
\end{aligned}
$$

A.4 O(+)H(+) Category

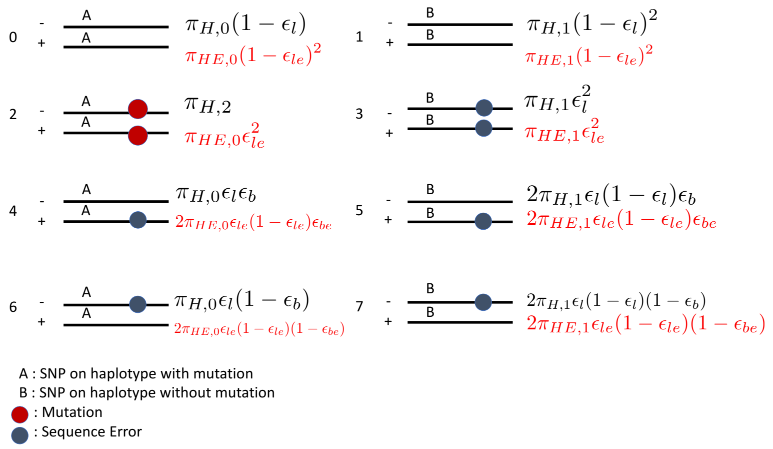

Figure 3:

Here we define functions reprepenting the logarithm of a paired-end read $r_{D,n}$ generation probability (where $t_{D,n} = 2$) given latent variable and parameters, as follows.

$$
\begin{aligned}
&L_{T,OH}(r_{D,n}, z^{(2)}_{D,n}, \pi_H, \epsilon_l, \epsilon_b) \\
&\quad := z^{(2)}_{D,n,0}\left\{\ln \pi_{H,0}(1-\epsilon_l) + \ln P(r_{D,n}|\mathcal{H}_{idx(z_{D,n,0})})\right\} \\
&\quad + z^{(2)}_{D,n,1}\left\{\ln \pi_{H,1}(1-\epsilon_l)^2 + \ln P(r_{D,n}|\mathcal{H}_{idx(z_{D,n,1})})\right\} \\
&\quad + z^{(2)}_{D,n,2}\left\{\ln \pi_{H,2} + \ln P(r_{D,n}|\mathcal{H}_{idx(z_{D,n,2})})\right\} \\
&\quad + z^{(2)}_{D,n,3}\left\{\ln \pi_{H,1}\epsilon_l^2 + \ln P(r_{D,n}|\mathcal{H}_{idx(z_{D,n,3})})\right\}
\end{aligned}
$$

$$+ z^{(2)}_{D,n,4}\left\{\ln \pi_{H,0}\epsilon_l\epsilon_b + \ln P(r_{D,n}|\mathcal{H}_{idx(z_{D,n,4})})\right\}$$
$$+ z^{(2)}_{D,n,5}\left\{\ln 2\pi_{H,1}(1-\epsilon_l)\epsilon_l\epsilon_b + \ln P(r_{D,n}|\mathcal{H}_{idx(z_{D,n,5})})\right\}$$
$$+ z^{(2)}_{D,n,6}\left\{\ln \pi_{H,0}\epsilon_l(1-\epsilon_b) + \ln P(r_{D,n}|\mathcal{H}_{idx(z_{D,n,6})})\right\}$$
$$+ z^{(2)}_{D,n,7}\left\{\ln 2\pi_{H,1}\epsilon_l(1-\epsilon_l)(1-\epsilon_b) + \ln P(r_{D,n}|\mathcal{H}_{idx(z_{D,n,7})})\right\}, D \in \{T, N\}$$

$$L_{E,OH}(r_{D,n}, z^{(2)}_{D,n}, \pi_{HE}, \epsilon_{le}, \epsilon_{be})$$
$$:= z^{(2)}_{D,n,0}\left\{\ln \pi_{HE,0}(1-\epsilon_{le})^2 + \ln P(r_{D,n}|\mathcal{H}_{idx(z^{(2)}_{D,n,0})})\right\}$$
$$+ z^{(2)}_{D,n,1}\left\{\ln \pi_{HE,1}(1-\epsilon_{le})^2 + \ln P(r_{D,n}|\mathcal{H}_{idx(z^{(2)}_{D,n,1})})\right\}$$
$$+ z^{(2)}_{D,n,2}\left\{\ln \pi_{HE,0}\epsilon_{le}^2 + \ln P(r_{D,n}|\mathcal{H}_{idx(z^{(2)}_{D,n,2})})\right\}$$
$$+ z^{(2)}_{D,n,3}\left\{\ln \pi_{HE,1}\epsilon_{le}^2 + \ln P(r_{D,n}|\mathcal{H}_{idx(z^{(2)}_{D,n,3})})\right\}$$
$$+ z^{(2)}_{D,n,4}\left\{\ln 2\pi_{HE,0}(1-\epsilon_{le})\epsilon_{le}\epsilon_{be} + \ln P(r_{D,n}|\mathcal{H}_{idx(z^{(2)}_{D,n,4})})\right\}$$
$$+ z^{(2)}_{D,n,5}\left\{\ln 2\pi_{HE,1}(1-\epsilon_{le})\epsilon_{le}\epsilon_{be} + \ln P(r_{D,n}|\mathcal{H}_{idx(z^{(2)}_{D,n,5})})\right\}$$
$$+ z^{(2)}_{D,n,6}\left\{\ln 2\pi_{HE,0}\epsilon_{le}(1-\epsilon_{le})(1-\epsilon_{be}) + \ln P(r_{D,n}|\mathcal{H}_{idx(z^{(2)}_{D,n,6})})\right\}$$
$$+ z^{(2)}_{D,n,7}\left\{\ln 2\pi_{HE,1}\epsilon_{le}(1-\epsilon_{le})(1-\epsilon_{be}) + \ln P(r_{D,n}|\mathcal{H}_{idx(z^{(2)}_{D,n,7})})\right\}, D \in \{T, N\}$$

A.5 O(-)H(-)S(+) Category

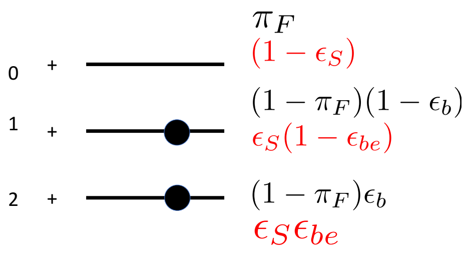

Figure 4:

Here we define functions reprepenting the logarithm of a paired-end read $r_{D,n}$ generation probability (where $t_{D,n} = 3$) given latent variable and parameters, as follows.

$$
\begin{aligned}
&L_{T,P}(r_{D,n}, z^{(3)}_{D,n}, \pi_F, \epsilon_b) \\
&:= z^{(3)}_{D,n,0}\left\{\ln \pi_F + \ln P(r_{D,n}|\mathcal{H}_{idx(z^{(3)}_{D,n,0})})\right\} \\
&\quad + z^{(3)}_{D,n,1}\left\{\ln(1-\pi_F)(1-\epsilon_b) + \ln P(r_{D,n}|\mathcal{H}_{idx(z^{(3)}_{D,n,1})})\right\} \\
&\quad + z^{(3)}_{D,n,2}\left\{\ln(1-\pi_F)\epsilon_b + \ln P(r_{D,n}|\mathcal{H}_{idx(z^{(3)}_{D,n,2})})\right\}, D \in \{T, N\}
\end{aligned}
$$

$$
\begin{aligned}
&L_{E,P}(r_{D,n}, z^{(3)}_{D,n}, \epsilon_S, \epsilon_{be}) \\
&\quad := z^{(3)}_{D,n,0}\left\{\ln(1-\epsilon_s) + \ln P(r_{D,n}|\mathcal{H}_{idx(z^{(3)}_{D,n,0})})\right\} \\
&\quad + z^{(3)}_{D,n,1}\left\{\ln \epsilon_s(1-\epsilon_{be}) + \ln P(r_{D,n}|\mathcal{H}_{idx(z^{(3)}_{D,n,1})})\right\} \\
&\quad + z^{(3)}_{D,n,2}\left\{\ln \epsilon_s\epsilon_{be} + \ln P(r_{D,n}|\mathcal{H}_{idx(z^{(3)}_{D,n,2})})\right\}, D \in \{T, N\}
\end{aligned}
$$

A.6 O(-)H(-)S(-) Category

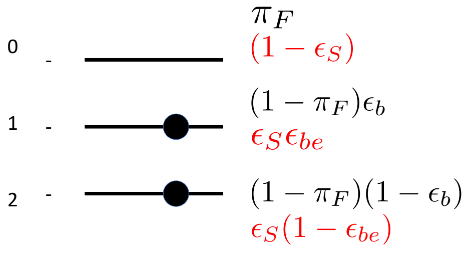

Figure 5:

Here we define functions reprepenting the logarithm of a paired-end read $r_{D,n}$ generation probability (where $t_{D,n} = 4$) given latent variable and parameters, as follows.

$$
\begin{aligned}
&L_{T,M}(r_{D,n}, z^{(4)}_{D,n}, \pi_F, \epsilon_b) \\
&\quad := z^{(4)}_{D,n,0}\left\{\ln \pi_F + \ln P(r_{D,n}|\mathcal{H}_{idx(z^{(4)}_{D,n,0})})\right\} \\
&\quad + z^{(4)}_{D,n,1}\left\{\ln(1-\pi_F)\epsilon_b + \ln P(r_{D,n}|\mathcal{H}_{idx(z^{(4)}_{D,n,1})})\right\} \\
&\quad + z^{(4)}_{D,n,2}\left\{\ln(1-\pi_F)(1-\epsilon_b) + \ln P(r_{D,n}|\mathcal{H}_{idx(z^{(4)}_{D,n,2})})\right\}, D \in \{T, N\}
\end{aligned}
$$

$$
\begin{aligned}
&L_{E,M}(r_{D,n}, z^{(4)}_{D,n}, \epsilon_S, \epsilon_{be}) \\
&\quad := z^{(4)}_{D,n,0}\left\{\ln(1-\epsilon_s) + \ln P(r_{D,n}|\mathcal{H}_{idx(z^{(4)}_{D,n,0})})\right\} \\
&\quad + z^{(4)}_{D,n,1}\left\{\ln \epsilon_s\epsilon_{be} + \ln P(r_{D,n}|\mathcal{H}_{idx(z^{(4)}_{D,n,1})})\right\} \\
&\quad + z^{(4)}_{D,n,2}\left\{\ln \epsilon_s(1-\epsilon_{be}) + \ln P(r_{D,n}|\mathcal{H}_{idx(z^{(4)}_{D,n,2})})\right\}, D \in \{T, N\}
\end{aligned}
$$

A.7 All the parameters and hyperparameters for tumor generative model in OHVarfinDer

Table 1: Notation summary

Notation	Type	Meaning
Δ^k	k dimensional non negative simplex	Definition of type
$\boldsymbol{\pi}_H$	Δ^3	A parameter for haplotype frequencies with variant
π_F	real number $\in [0,1]$	A parameter for reference allele frequency
ϵ_l	real number $\in [0,1]$	A parameter for error rate in overlapping paired-end reads
ϵ_h	real number $\in [0,1]$	A parameter for error rate in hetero SNP covering reads
ϵ_b	real number $\in [0,1]$	A parameter for strand bias rate
π_{HE}	real number $\in [0,1]$	A haplotype frequency without variant
ϵ_s	real number $\in [0,1]$	An error rate for unpaired read
$\boldsymbol{\gamma}_H$	$(\mathbb{R}_+, \mathbb{R}_+, \mathbb{R}_+)$	A hyperparameter for $\boldsymbol{\pi}_H$
$\boldsymbol{\gamma}_F$	$(\mathbb{R}_+, \mathbb{R}_+)$	A hyperparameter for $\boldsymbol{\pi}_F$
$\boldsymbol{\alpha}_l$	$(\mathbb{R}_+, \mathbb{R}_+)$	A hyperparameter for ϵ_l
$\boldsymbol{\alpha}_h$	$(\mathbb{R}_+, \mathbb{R}_+)$	A hyperparameter for ϵ_h
$\boldsymbol{\alpha}_b$	$(\mathbb{R}_+, \mathbb{R}_+)$	A hyperparameter for ϵ_b
$\boldsymbol{\gamma}_{HE}$	$(\mathbb{R}_+, \mathbb{R}_+)$	A hyperparameter for $\boldsymbol{\pi}_{HE}$
$\boldsymbol{\alpha}_s$	$(\mathbb{R}_+, \mathbb{R}_+)$	A hyperparameter for ϵ_s

A.8 Joint Probability for Tumor Generative Model

$$\begin{aligned}
&\ln Pr(\mathcal{R}_{NT}, \mathcal{Z}_{T,NT} | \mathcal{M}_T, \{t_n\}_n) \\
&= \ln P_{bata}(\boldsymbol{\pi}_{HE}|\boldsymbol{\gamma}_{HE}) + \ln P_{dir}(\boldsymbol{\pi}_H|\boldsymbol{\gamma}_H) + \ln P_{beta}(\boldsymbol{\pi}_F|\boldsymbol{\gamma}_F) \\
&\quad + \ln P_{bata}(\epsilon_s|\boldsymbol{\alpha}_s) + \ln P_{bata}(\epsilon_l|\boldsymbol{\alpha}_l) + \ln P_{bata}(\epsilon_h|\boldsymbol{\alpha}_h) + \ln P_{bata}(\epsilon_b|\boldsymbol{\alpha}_b) \\
&\quad + \sum_{n|t_{T,n}=0} L_{T,O}(r_{T,n}, z^{(0)}_{T,n}, \pi_F, \epsilon_l, \epsilon_b) \\
&\quad + \sum_{n|t_{T,n}=1} L_{T,H}(r_{T,n}, z^{(1)}_{T,n}, \boldsymbol{\pi}_H, \epsilon_h) \\
&\quad + \sum_{n|t_{T,n}=2} L_{T,OH}(r_{T,n}, z^{(2)}_{T,n}, \pi_H, \epsilon_l, \epsilon_b) \\
&\quad + \sum_{n|t_{T,n}=3} L_{T,P}(r_{T,n}, z^{(3)}_{T,n}, \pi_F, \epsilon_b) \\
&\quad + \sum_{n|t_{T,n}=4} L_{T,M}(r_{T,n}, z^{(4)}_{T,n}, \pi_F, \epsilon_b) \\
&\quad + \sum_{n|t_{N,n}=0} L_{E,O}(r_{N,n}, z^{(0)}_{N,n}, \epsilon_l, \epsilon_b) \\
&\quad + \sum_{n|t_{N,n}=1} L_{E,H}(r_{N,n}, z^{(1)}_{N,n}, \pi_{HE}, \epsilon_h) \\
&\quad + \sum_{n|t_{N,n}=2} L_{E,OH}(r_{N,n}, z^{(2)}_{N,n}, \pi_{HE}, \epsilon_l, \epsilon_b)
\end{aligned}$$

$$+ \sum_{n|t_{N,n}=3} L_{E,P}(r_{N,n}, z^{(3)}_{N,n}, \epsilon_S, \epsilon_b)$$
$$+ \sum_{n|t_{N,n}=4} L_{E,M}(r_{N,n}, z^{(4)}_{N,n}, \epsilon_S, \epsilon_b)$$

A.9 Assumptions on Variational Distributions

We assume fully independence on all the variational distribution as follows.

$$q(\mathcal{Z}_{S,NT}) = q(\boldsymbol{\theta}_{S,all}) \prod_{D\in\{T,N\}} \prod_{k=0}^{4} \prod_{n|t_n=k} q(z^{(k)}_{D,n})$$
$$q(\boldsymbol{\theta}_{S,all}) = q(\boldsymbol{\pi}_H)q(\pi_F)q(\epsilon_l)q(\epsilon_h)q(\epsilon_b)q(\pi_{HE})q(\epsilon_{be})q(\epsilon_S)$$

A.10 Lower Bound for Marginal Likelihoood in Tumor Generative Model

We denote the following lower bound as $\mathcal{L}_T(q)$ and we aim to maximize this lower bound with respect to each variational distribution.

$$\ln Pr(\mathcal{R}_{NT}|\mathcal{M}_S, \{t_n\}_n)$$
$$\geq E_q\left[\ln \frac{Pr(\mathcal{R}_{NT}, \mathcal{Z}_{S,NT}|\mathcal{M}_S, \{t_n\}_n)}{q(\mathcal{Z}_{S,NT})}\right] =: \mathcal{L}_T(q)$$

A.11 Maximize $\mathcal{L}_T(q)$ with respect to $q(\boldsymbol{\pi}_H)$

We would like to get optimal $q^*(\boldsymbol{\pi}_H)$ which maximize $\mathcal{L}_T(q)$ with respect to $q(\boldsymbol{\pi}_H)$.

$$\mathcal{L}_T(q)$$
$$= E_q\left[\left\{(\gamma_{H,0}-1) + \sum_n \left\{z^{(1)}_{T,n,0} + z^{(2)}_{T,n,0} + z^{(2)}_{T,n,4} + z^{(2)}_{T,n,6}\right\}\right\} \ln \pi_{H,0}\right]$$
$$+ E_q\left[\left\{(\gamma_{H,1}-1) + \sum_n \left\{z^{(1)}_{T,n,5} + z^{(1)}_{T,n,4} + z^{(2)}_{T,n,1} + z^{(2)}_{T,n,3} + z^{(2)}_{T,n,5} + z^{(2)}_{T,n,7}\right\}\right\} \ln \pi_{H,1}\right]$$
$$+ E_q\left[\left\{(\gamma_{H,2}-1) + \sum_n \left\{z^{(1)}_{T,n,2} + z^{(2)}_{T,n,2}\right\}\right\} \ln \pi_{H,2}\right]$$
$$- E_q\left[\ln q(\boldsymbol{\pi}_H)\right] + Const.$$
$$= -KL[q(\boldsymbol{\pi}_H)||p_{dir}(\boldsymbol{\pi}_H|\boldsymbol{\gamma}^*_H)] + Const.,$$

where

$$\gamma^*_{H,0} = E_q\left[(\gamma_{H,0}-1)+\sum_n\left\{z^{(1)}_{T,n,0}+z^{(2)}_{T,n,0}+z^{(2)}_{T,n,4}+z^{(2)}_{T,n,6}\right\}\right]$$

$$\gamma^*_{H,1} = E_q\left[(\gamma_{H,1}-1)+\sum_n\left\{z^{(1)}_{T,n,5}+z^{(1)}_{T,n,4}+z^{(2)}_{T,n,1}+z^{(2)}_{T,n,3}+z^{(2)}_{T,n,5}+z^{(2)}_{T,n,7}\right\}\right]$$

$$\gamma^*_{H,2} = E_q\left[(\gamma_{H,2}-1)+\sum_n\left\{z^{(1)}_{T,n,2}+z^{(2)}_{T,n,2}\right\}\right]$$

Therefore, we can maximize the lower bound by minimization of KL divergence of $KL[q(\boldsymbol{\pi}_H)||p_{dir}(\boldsymbol{\pi}_H|\boldsymbol{\gamma}^*_H)] \geq 0$. The optimal form distribution is

$$q(\boldsymbol{\pi}_H) = p_{dir}(\boldsymbol{\pi}_H|\boldsymbol{\gamma}^*_H)$$

A.12 Maximize $\mathcal{L}_T(q)$ with respect to $q(\boldsymbol{\pi}_F)$

We would like to get optimal $q^*(\pi_F)$ which maximize $\mathcal{L}_T(q)$ with respect to $q(\pi_F)$.

$$\begin{aligned}
&\mathcal{L}_T(q)\\
&= E_q\left[\left\{(\gamma_{F,0}-1)+\sum_n\left\{z^{(0)}_{T,n,0}+z^{(0)}_{T,n,2}+z^{(0)}_{T,n,3}+z^{(3)}_{T,n,0}+z^{(4)}_{T,n,0}\right\}\right\}\ln\pi_F\right]\\
&\quad + E_q\left[\left\{(\gamma_{F,1}-1)+\sum_n\left\{z^{(0)}_{T,n,1}+z^{(3)}_{T,n,1}+z^{(3)}_{T,n,2}+z^{(4)}_{T,n,1}+z^{(4)}_{T,n,2}\right\}\right\}\ln(1-\pi_F)\right]\\
&\quad - E_q\left[\ln q(\boldsymbol{\pi}_F)\right] + Const.\\
&= -KL[q(\pi_F)||p_{beta}(\pi_F|\boldsymbol{\gamma}^*_F)] + Const.,
\end{aligned}$$

where

$$\gamma^*_{F,0} = E_q\left[(\gamma_{F,0}-1)+\sum_n\left\{z^{(0)}_{T,n,0}+z^{(0)}_{T,n,2}+z^{(0)}_{T,n,3}+z^{(3)}_{T,n,0}+z^{(4)}_{T,n,0}\right\}\right]$$

$$\gamma^*_{F,1} = E_q\left[(\gamma_{F,1}-1)+\sum_n\left\{z^{(0)}_{T,n,1}+z^{(3)}_{T,n,1}+z^{(3)}_{T,n,2}+z^{(4)}_{T,n,1}+z^{(4)}_{T,n,2}\right\}\right]$$

Therefore, we can maximize the lower bound by minimization of KL divergence of $KL[q(\pi_H)||p_{beta}(\pi_F|\boldsymbol{\gamma}^*_F)] \geq 0$. The optimal form distribution is

$$q^*(\pi_F) = p_{beta}(\pi_F|\boldsymbol{\gamma}^*_F)$$

A.13 Maximize $\mathcal{L}_T(q)$ with respect to $q(\epsilon_l)$

We would like to get optimal $q^*(\epsilon_l)$ which maximize $\mathcal{L}_T(q)$ with respect to $q(\epsilon_l)$.

$$
\begin{aligned}
&\mathcal{L}_T(q) \\
&= E_q\left[\{(\alpha_{l,0}-1)\}\ln\epsilon_l\right] \\
&\quad + E_q\left[\left\{\sum_n\left\{z^{(0)}_{T,n,2}+z^{(0)}_{T,n,3}+2z^{(2)}_{T,n,3}+z^{(2)}_{T,n,4}+z^{(2)}_{T,n,5}+z^{(2)}_{T,n,6}+z^{(2)}_{T,n,7}\right\}\right\}\ln\epsilon_l\right] \\
&\quad + E_q\Bigg[\sum_n\Big\{2z^{(0)}_{N,n,1}+z^{(0)}_{N,n,2}+z^{(0)}_{N,n,3} \\
&\qquad\quad +2z^{(2)}_{N,n,2}+2z^{(2)}_{N,n,3}+z^{(2)}_{N,n,4}+z^{(2)}_{N,n,5}+z^{(2)}_{N,n,6}+z^{(2)}_{N,n,7}\Big\}\ln\epsilon_l\Bigg] \\
&\quad + E_q\left[\{(\alpha_{l,1}-1)\}\ln(1-\epsilon_l)\right] \\
&\quad + E_q\left[\sum_n\left\{z^{(0)}_{T,n,0}+z^{(2)}_{T,n,0}+2z^{(2)}_{T,n,1}+z^{(2)}_{T,n,5}+z^{(2)}_{T,n,7}\right\}\ln(1-\epsilon_l)\right] \\
&\quad + E_q\Bigg[\sum_n\Big\{2z^{(0)}_{N,n,0}+z^{(0)}_{N,n,2}+z^{(0)}_{N,n,3} \\
&\qquad\quad +2z^{(2)}_{N,n,0}+2z^{(2)}_{N,n,1}+z^{(2)}_{N,n,4}+z^{(2)}_{N,n,5}+z^{(2)}_{N,n,6}+z^{(2)}_{N,n,7}\Big\}\ln(1-\epsilon_l)\Bigg] \\
&\quad - E_q\left[\ln q(\boldsymbol{\epsilon}_l)\right]+Const. \\
&= -KL[q(\epsilon_l)||p_{beta}(\epsilon_l|\boldsymbol{\alpha}^*_l)]+Const.,
\end{aligned}
$$

where

$$
\begin{aligned}
\alpha^*_{l,0} &= (\alpha_{h,0}-1)+\sum_n E_q\left[z^{(0)}_{T,n,2}+z^{(0)}_{T,n,3}+2z^{(2)}_{T,n,3}\right] \\
&\quad +\sum_n E_q\left[z^{(2)}_{T,n,4}+z^{(2)}_{T,n,5}+z^{(2)}_{T,n,6}+z^{(2)}_{T,n,7}\right] \\
&\quad +\sum_n E_q\left[2z^{(0)}_{N,n,1}+z^{(0)}_{N,n,2}+z^{(0)}_{N,n,3}\right] \\
&\quad +\sum_n E_q\left[2z^{(2)}_{N,n,2}+2z^{(2)}_{N,n,3}+z^{(2)}_{N,n,4}+z^{(2)}_{N,n,5}+z^{(2)}_{N,n,6}\right] \\
\alpha^*_{l,1} &= (\alpha_{h,1}-1)+\sum_n E_q\left[z^{(0)}_{T,n,0}+z^{(2)}_{T,n,0}+2z^{(2)}_{T,n,1}+z^{(2)}_{T,n,5}+z^{(2)}_{T,n,7}\right] \\
&\quad +\sum_n E_q\left[z^{(2)}_{T,n,4}+z^{(2)}_{T,n,5}+z^{(2)}_{T,n,6}+z^{(2)}_{T,n,7}\right] \\
&\quad +\sum_n E_q\left[2z^{(0)}_{N,n,0}+z^{(0)}_{N,n,2}+z^{(0)}_{N,n,3}\right]
\end{aligned}
$$

$$+\sum_n E_q\left[2z^{(2)}_{N,n,0}+2z^{(2)}_{N,n,1}+z^{(2)}_{N,n,4}+z^{(2)}_{N,n,5}+z^{(2)}_{N,n,6}+z^{(2)}_{N,n,7}\right]$$

Therefore, we can maximize the lower bound by minimization of KL divergence of $KL[q(\epsilon_l)||p_{beta}(\epsilon_l|\boldsymbol{\alpha}^*_l)]\geq 0$. The optimal form distribution is

$$q^*(\epsilon_l)=p_{beta}(\epsilon_l|\boldsymbol{\alpha}^*_l)$$

A.14 Maximize $\mathcal{L}_T(q)$ with respect to $q(\epsilon_h)$

We would like to get optimal $q^*(\epsilon_h)$ which maximize $\mathcal{L}_T(q)$ with respect to $q(\epsilon_h)$.

$$\begin{aligned}\mathcal{L}_T(q)=&E_q\left[\left\{(\alpha_{l,0}-1)+\sum_n\left\{z^{(1)}_{T,n,3}\right\}\right\}\ln\epsilon_h\right]\\&+E_q\left[\sum_n\left\{z^{(1)}_{N,n,2}+z^{(1)}_{N,n,3}\right\}\ln\epsilon_h\right]\\&+E_q\left[\left\{(\alpha_{l,1}-1)+\sum_n z^{(1)}_{T,n,1}\right\}\ln(1-\epsilon_h)\right]\\&+E_q\left[\sum_n\left\{z^{(1)}_{N,n,0}+z^{(1)}_{N,n,1}\right\}\ln(1-\epsilon_h)\right]\\&-E_q\left[\ln q(\boldsymbol{\epsilon}_h)\right]+Const.\\=&-KL[q(\epsilon_h)||p_{beta}(\epsilon_h|\boldsymbol{\alpha}^*_h)]+Const.,\end{aligned}$$

where

$$\begin{aligned}\alpha^*_{h,0}&=E_q\left[(\alpha_{h,0}-1)+\sum_n\left\{z^{(1)}_{T,n,3}\right\}+\sum_n\left\{z^{(1)}_{N,n,2}+z^{(1)}_{N,n,3}\right\}\right]\\\alpha^*_{h,1}&=E_q\left[(\alpha_{h,1}-1)+\sum_n z^{(1)}_{T,n,1}+\sum_n\left\{z^{(1)}_{N,n,0}+z^{(1)}_{N,n,1}\right\}\right]\end{aligned}$$

Therefore, we can maximize the lower bound by minimization of KL divergence of $KL[q(\epsilon_h)||p_{beta}(\epsilon_h|\boldsymbol{\alpha}^*_h)]$. The optimal form distribution is

$$q^*(\epsilon_h)=p_{beta}(\epsilon_h|\boldsymbol{\alpha}^*_h)$$

A.15 Maximize $\mathcal{L}_T(q)$ with respect to $q(\epsilon_b)$

We would like to get optimal $q^*(\epsilon_b)$ which maximize $\mathcal{L}_T(q)$ with respect to $q(\epsilon_b)$.

$$
\begin{aligned}
\mathcal{L}_T(q) &= E_q\left[\left\{(\alpha_{b,0}-1)+\sum_D\sum_n\left\{z^{(0)}_{D,n,2}+z^{(2)}_{D,n,4}+z^{(2)}_{D,n,5}+z^{(3)}_{D,n,2}+z^{(4)}_{D,n,1}\right\}\right\}\ln\epsilon_b\right]\\
&\quad+E_q\left[\left\{(\alpha_{b,1}-1)+\sum_D\sum_n\left\{z^{(0)}_{D,n,3}+z^{(2)}_{D,n,6}+z^{(2)}_{D,n,7}+z^{(3)}_{D,n,1}+z^{(4)}_{D,n,2}\right\}\right\}\ln(1-\epsilon_b)\right]\\
&\quad-E_q\left[\ln q(\boldsymbol{\epsilon}_b)\right]+Const.\\
&= -KL[q(\epsilon_b)||p_{beta}(\epsilon_b|\boldsymbol{\alpha}^*_b)]+Const.,
\end{aligned}
$$

where

$$
\begin{aligned}
\alpha^*_{b,0} &= E_q\left[(\alpha_{b,0}-1)+\sum_D\sum_n\left\{z^{(0)}_{D,n,2}+z^{(2)}_{D,n,4}+z^{(2)}_{D,n,5}+z^{(3)}_{D,n,2}+z^{(4)}_{D,n,1}\right\}\right]\\
\alpha^*_{b,1} &= E_q\left[(\alpha_{b,1}-1)+\sum_D\sum_n\left\{z^{(0)}_{D,n,3}+z^{(2)}_{D,n,6}+z^{(2)}_{D,n,7}+z^{(3)}_{D,n,1}+z^{(4)}_{D,n,2}\right\}\right]
\end{aligned}
$$

Therefore, we can maximize the lower bound by minimization of KL divergence of $KL[q(\epsilon_b)||p_{beta}(\epsilon_b|\boldsymbol{\alpha}^*_b)]\geq 0$. The optimal form distribution is

$$
q^*(\epsilon_b)=p_{beta}(\epsilon_b|\boldsymbol{\alpha}^*_b)
$$

A.16 Maximize $\mathcal{L}_T(q)$ with respect to $q(\boldsymbol{z}^{(k)}_{D,n})$

In order to show updating procedure w.r.t $q(\boldsymbol{z}^{(k)}_{D,n})$ for $k\in\{0,1,2,3,4\}, D\in\{T,N\}$, it is enough to show the procedure w.r.t $q(\boldsymbol{z}^{(0)}_{T,n})$.

$$
\begin{aligned}
\mathcal{L}_T(q) &= E_q\left[z^{(0)}_{T,n,0}\right]E_q\left[\ln\pi_F(1-\epsilon_l)+\ln P(r_{T,n}|\mathcal{H}_{idx(z^{(0)}_{T,n,0})})\right]\\
&\quad+E_q\left[z^{(0)}_{T,n,1}\right]E_q\left[\ln(1-\pi_F)+\ln P(r_{T,n}|\mathcal{H}_{idx(z^{(0)}_{T,n,1})})\right]\\
&\quad+E_q\left[z^{(0)}_{T,n,2}\right]E_q\left[\ln\pi_F\epsilon_l\epsilon_b+\ln P(r_{T,n}|\mathcal{H}_{idx(z^{(0)}_{T,n,2})})\right]\\
&\quad+E_q\left[z^{(0)}_{T,n,3}\right]E_q\left[\ln\pi_F\epsilon_l(1-\epsilon_b)+\ln P(r_{T,n}|\mathcal{H}_{idx(z^{(0)}_{T,n,3})})\right]\\
&\quad-E_q\left[\ln q(\boldsymbol{z}^{(0)}_{T,n})\right]+Const.\\
&= E_q\left[z^{(0)}_{T,n,0}\right]E_q\left[\ln\rho^*_{T,n,0}\right]+E_q\left[z^{(0)}_{T,n,1}\right]E_q\left[\ln\rho^*_{T,n,1}\right]\\
&\quad+E_q\left[z^{(0)}_{T,n,2}\right]E_q\left[\ln\rho^*_{T,n,2}\right]+E_q\left[z^{(0)}_{T,n,3}\right]E_q\left[\ln\rho^*_{T,n,3}\right]\\
&\quad-E_q\left[\ln q(\boldsymbol{z}^{(0)}_{T,n})\right]+Const.\\
&= -KL[q(\boldsymbol{z}^{(0)}_{T,n})||p_{multi}(\boldsymbol{z}^{(0)}_{T,n}|\boldsymbol{\zeta}^*_{T,n})]+Const.,
\end{aligned}
$$

where

$$\zeta^*_{T,n,j} \propto \rho^*_{T,n,j}$$
$$\sum_{j=0}^{3} \zeta^*_{T,n,j} = 1$$

Therefore, we can maximize the lower bound by minimization of KL divergence of $KL[q(\boldsymbol{z}^{(0)}_{T,n})||p_{multi}(\boldsymbol{z}^{(0)}_{T,n}|\boldsymbol{\zeta}^*_{T,n})] \geq 0$. The optimal form distribution is

$$q(\boldsymbol{z}^{(0)}_{T,n}) = p_{multi}(\boldsymbol{z}^{(0)}_{T,n}|\boldsymbol{\zeta}^*_{T,n})$$

A.17 All the Parameters and Hyperparameters for Error Generative Model in OHVarfinDer

Table 2: Notation summary

Notation	Type	Meaning
Δ^k	k dimensional non negative simplex	Definition of type
$\pi_{HE}, \pi_{T,HE}, \pi_{N,HE}$	real number $\in [0,1]$	Parameters for haplotype frequencies with variant
$\epsilon_l, \epsilon_{T,l}, \epsilon_{N,l}$	real number $\in [0,1]$	Parameters for error rate in overlapping paired-end reads
$\epsilon_h, \epsilon_{T,h}, \epsilon_{N,h}$	real number $\in [0,1]$	Parameters for error rate in hetero SNP covering reads
$\epsilon_b, \epsilon_{T,b}, \epsilon_{N,b}$	real number $\in [0,1]$	Parameters for strand bias rate
$\epsilon_s, \epsilon_{T,s}, \epsilon_{N,s}$	real number $\in [0,1]$	Error rates for unpaired read
$\boldsymbol{\gamma}_{HE}, \boldsymbol{\gamma}_{T,HE}, \boldsymbol{\gamma}_{N,HE}$	$(\mathbb{R}_+, \mathbb{R}_+)$	Hyperparameters for $\boldsymbol{\pi}_{HE}$
$\boldsymbol{\alpha}_l, \boldsymbol{\alpha}_{T,l}, \boldsymbol{\alpha}_{N,l}$	$(\mathbb{R}_+, \mathbb{R}_+)$	Hyperparameters for ϵ_l
$\boldsymbol{\alpha}_h, \boldsymbol{\alpha}_{T,h}, \boldsymbol{\alpha}_{N,h}$	$(\mathbb{R}_+, \mathbb{R}_+)$	Hyperparameters for ϵ_h
$\boldsymbol{\alpha}_b, \boldsymbol{\alpha}_{T,b}, \boldsymbol{\alpha}_{N,b}$	$(\mathbb{R}_+, \mathbb{R}_+)$	Hyperparameters for ϵ_b
$\boldsymbol{\alpha}_s, \boldsymbol{\alpha}_{T,s}, \boldsymbol{\alpha}_{N,s}$	$(\mathbb{R}_+, \mathbb{R}_+)$	Hyperparameters for ϵ_s

A.18 Different Error Generative Models for Whole and Exome Data Sets

In contrast to the whole genome data sets, it is rare that variant supporting reads with relatively low base quality (between 10 to 15) appears both in normal and tumor exome sequence data. The reason comes from the filter condition for collecting the candidate somatic mutations.

Firstly, we assume the following erroneous variant supporting generation process. Let p_v is the probability of the erroneous variant supporting reads generation in normal sequence data. Let $p_{b,v}$ is the probability that low base quality (less than 15) appears on a variant supporting read. Secondly, we assume the following filter condition: if the number of variant supporting read with higher base quality in normal sequence data is ≥ 2, we filter the candidate mutation position.

Under these two assumptions, one read is variant supporting and with high base quality (≥ 15) with probability of $p_v(1 - p_{b,v})$. If in a erroneous position, $p_v = 0.1, (1 - p_{b,v}) = 0.5$ and $p_v(1 - p_{b,v}) = 0.05$, and depth in normal data is about 200, it is a rare event to pass the above filter condition; average number of variant supporting read with higher base quality is 10 if depth is 200. If depth is about 30, it is not a rare event to pass the above filter condition; average number of variant supporting read with higher base quality is 1.5 if depth is 30.

Therefore, the distributions of sequence data should be different if depth condition is different. We modeled the error generative model differently between exome sequence data and whole genome data.

A.19 Joint Probability for Error Generative Model in Whole Genoeme Sequence Data

In whole genome sequence data sets, variant supporting reads with relatively low base quality (between 10 to 15) often appear both in normal sequence data and tumor sequence data. In order to treat these situation, we modeled that parameter is shared between tumor and normal sequnce data as follows. The variational Bayes procedures are similar to the case in tumor generative model.

$$
\begin{aligned}
&\ln Pr(\mathcal{R}_{NT}, \mathcal{Z}_{E,NT} | \mathcal{M}_E, \{t_n\}_n) \\
&= \ln P_{bata}(\boldsymbol{\pi}_{HE} | \boldsymbol{\gamma}_{HE}) + \ln P_{bata}(\epsilon_s | \boldsymbol{\alpha}_s) \\
&\quad + \ln P_{bata}(\epsilon_l | \boldsymbol{\alpha}_l) + \ln P_{bata}(\epsilon_h | \boldsymbol{\alpha}_h) + \ln P_{bata}(\epsilon_b | \boldsymbol{\alpha}_b) \\
&\quad + \sum_{n|t_{T,n}=0} L_{E,O}(r_{T,n}, z^{(0)}_{T,n}, \epsilon_l, \epsilon_b) + \sum_{n|t_{T,n}=1} L_{E,H}(r_{T,n}, z^{(1)}_{T,n}, \pi_{HE}, \epsilon_h) \\
&\quad + \sum_{n|t_{T,n}=2} L_{E,OH}(r_{T,n}, z^{(2)}_{T,n}, \pi_{HE}, \epsilon_l, \epsilon_b) + \sum_{n|t_{T,n}=3} L_{E,P}(r_{T,n}, z^{(3)}_{T,n}, \epsilon_S, \epsilon_b) \\
&\quad + \sum_{n|t_{T,n}=4} L_{E,M}(r_{T,n}, z^{(4)}_{T,n}, \epsilon_S, \epsilon_b) \\
&\quad + \sum_{n|t_{N,n}=0} L_{E,O}(r_{N,n}, z^{(0)}_{N,n}, \epsilon_l, \epsilon_b) + \sum_{n|t_{N,n}=1} L_{E,H}(r_{N,n}, z^{(1)}_{N,n}, \pi_{HE}, \epsilon_h) \\
&\quad + \sum_{n|t_{N,n}=2} L_{E,OH}(r_{N,n}, z^{(2)}_{N,n}, \pi_{HE}, \epsilon_l, \epsilon_b) + \sum_{n|t_{N,n}=3} L_{E,P}(r_{N,n}, z^{(3)}_{N,n}, \epsilon_S, \epsilon_b) \\
&\quad + \sum_{n|t_{N,n}=4} L_{E,M}(r_{N,n}, z^{(4)}_{N,n}, \epsilon_S, \epsilon_b)
\end{aligned}
$$

A.20 Joint Probability For Error Generative Model in Exome Sequence Data

In exome sequence data sets, variant supporting reads with relatively low base quality (between 10 to 15) do not appear both in normal sequence data and tumor sequence data. In order to treat these situation, we modeled that parameter is not shared between tumor and normal sequnce data as follows. The variational Bayes procedures are similar to the case in tumor generative model.

$$
\begin{aligned}
&\ln Pr(\mathcal{R}_{NT}, \mathcal{Z}_{E,NT} | \mathcal{M}_E, \{t_n\}_n) \\
&= \ln P_{bata}(\boldsymbol{\pi}_{T,HE} | \gamma_{T,HE}) + \ln P_{bata}(\epsilon_{T,s} | \boldsymbol{\alpha}_{T,s}) \\
&\quad + \ln P_{bata}(\epsilon_{T,l} | \boldsymbol{\alpha}_{T,l}) + \ln P_{bata}(\epsilon_{T,h} | \boldsymbol{\alpha}_{T,h}) + \ln P_{bata}(\epsilon_{T,b} | \boldsymbol{\alpha}_{T,b}) \\
&\quad + \ln P_{bata}(\boldsymbol{\pi}_{N,HE} | \gamma_{N,HE}) + \ln P_{bata}(\epsilon_{N,s} | \boldsymbol{\alpha}_{N,s}) \\
&\quad + \ln P_{bata}(\epsilon_{N,l} | \boldsymbol{\alpha}_{N,l}) + \ln P_{bata}(\epsilon_{N,h} | \boldsymbol{\alpha}_{N,h}) + \ln P_{bata}(\epsilon_{N,b} | \boldsymbol{\alpha}_{N,b}) \\
&\quad + \sum_{n|t_{T,n}=0} L_{E,O}(r_{T,n}, z^{(0)}_{T,n}, \epsilon_{T,l}, \epsilon_{T,b}) + \sum_{n|t_{T,n}=1} L_{E,H}(r_{T,n}, z^{(1)}_{T,n}, \pi_{T,HE}, \epsilon_{T,h}) \\
&\quad + \sum_{n|t_{T,n}=2} L_{E,OH}(r_{T,n}, z^{(2)}_{T,n}, \pi_{T,HE}, \epsilon_{T,l}, \epsilon_{T,b}) + \sum_{n|t_{T,n}=3} L_{E,P}(r_{T,n}, z^{(3)}_{T,n}, \epsilon_{T,S}, \epsilon_{T,b}) \\
&\quad + \sum_{n|t_{T,n}=4} L_{E,M}(r_{T,n}, z^{(4)}_{T,n}, \epsilon_{T,S}, \epsilon_{T,b}) \\
&\quad + \sum_{n|t_{N,n}=0} L_{E,O}(r_{N,n}, z^{(0)}_{N,n}, \epsilon_{N,l}, \epsilon_{N,b}) + \sum_{n|t_{N,n}=1} L_{E,H}(r_{N,n}, z^{(1)}_{N,n}, \pi_{N,HE}, \epsilon_{N,h}) \\
&\quad + \sum_{n|t_{N,n}=2} L_{E,OH}(r_{N,n}, z^{(2)}_{N,n}, \pi_{N,HE}, \epsilon_{N,l}, \epsilon_{N,b}) + \sum_{n|t_{N,n}=3} L_{E,P}(r_{N,n}, z^{(3)}_{N,n}, \epsilon_{N,S}, \epsilon_{N,b}) \\
&\quad + \sum_{n|t_{N,n}=4} L_{E,M}(r_{N,n}, z^{(4)}_{N,n}, \epsilon_{N,S}, \epsilon_{N,b})
\end{aligned}
$$

A.21 Prior Hyperparameters Used in Performance Evaluation

Table 3: Prior hyperparameters summary

Experiment	Model	Hyperparameters	Depth < 100	Depth ≥ 100
Simulation	Error model	$\gamma_{HE}, \gamma_{T,HE}, \gamma_{N,HE}$	$(5.0, 5.0)$	”
		$\boldsymbol{\alpha}_l, \boldsymbol{\alpha}_{T,l}, \boldsymbol{\alpha}_{N,l}$	$(2.0, 30.0)$	”
		$\boldsymbol{\alpha}_h, \boldsymbol{\alpha}_{T,h}, \boldsymbol{\alpha}_{N,h}$	$(2.0, 30.0)$	”
		$\boldsymbol{\alpha}_b, \boldsymbol{\alpha}_{T,b}, \boldsymbol{\alpha}_{N,b}$	$(0.5, 0.5)$	$(0.05, 0.05)$
		$\boldsymbol{\alpha}_s, \boldsymbol{\alpha}_{T,s}, \boldsymbol{\alpha}_{N,s}$	$(2.0, 30.0)$	”
	Tumor model	$\boldsymbol{\gamma}_H$	$(5.0, 5.0, 1.0)$	”
		γ_{HE}	$(5.0, 5.0)$	”
		$\boldsymbol{\gamma}_F$	$(10.0, 1.0)$	”
		$\boldsymbol{\alpha}_l$	$(1.0, 100.0)$	”
		$\boldsymbol{\alpha}_h$	$(1.0, 100.0)$	”
		$\boldsymbol{\alpha}_s$	$(1.0, 100.0)$	”
		$\boldsymbol{\alpha}_b$	$(1.0, 1.0)$	$(10.0, 10.0)$
Real data	Error model	$\gamma_{HE}, \gamma_{T,HE}, \gamma_{N,HE}$	$(1.0, 1.0)$	”
		$\boldsymbol{\alpha}_l, \boldsymbol{\alpha}_{T,l}, \boldsymbol{\alpha}_{N,l}$	$(1.0, 10.0)$	”
		$\boldsymbol{\alpha}_h, \boldsymbol{\alpha}_{T,h}, \boldsymbol{\alpha}_{N,h}$	$(1.0, 10.0)$	”
		$\boldsymbol{\alpha}_b, \boldsymbol{\alpha}_{T,b}, \boldsymbol{\alpha}_{N,b}$	$(0.2, 0.2)$	$(0.05, 0.05)$
		$\boldsymbol{\alpha}_s, \boldsymbol{\alpha}_{T,s}, \boldsymbol{\alpha}_{N,s}$	$(1.0, 10.0)$	”
	Tumor model	$\boldsymbol{\gamma}_H$	$(2.5, 2.5, 1.0)$	”
		γ_{HE}	$(1.0, 1.0)$	”
		$\boldsymbol{\gamma}_F$	$(5.0, 1.0)$	”
		$\boldsymbol{\alpha}_l$	$(0.1, 10.0)$	”
		$\boldsymbol{\alpha}_h$	$(0.1, 10.0)$	”
		$\boldsymbol{\alpha}_s$	$(0.1, 10.0)$	”
		$\boldsymbol{\alpha}_b$	$(5.0, 5.0)$	$(10.0, 10.0)$

As for the hyperparameters for simulation data sets, we refer to the data generation procedure in the OVarCall experiment, we set the hyperparameters based on the hyperparameters in OVarCall. As for the real data sets (especially whole genome data sets) we used the same data sets used in HapMuC, therefore we refer to the HapMuC in the setting of the hyperparameters.

B Details of Result

B.1 Filter Conditions for Simulation Data Sets

We retained the candidate positions if they met with the following criteria.

1. The read coverage is ≥ 12
2. The non-reference allele frequency in tumor sample is ≥ 0.05
3. The normal allele frequency in normal sample is ≤ 0.1
4. Variant supporting read in tumor sample is ≥ 4.

B.2 Filter Conditions for Exome Sequence Data

We retained the candidate positions for lower variant allele frequency mutations if they met with the following criteria.

1. The read coverage is ≥ 100
2. The non-reference allele frequency in tumor sample is ≥ 0.02 and ≤ 0.07.
3. The normal allele frequency in normal sample is ≤ 0.01
4. Variant supporting read in tumor sample is ≥ 3.
5. Variant supporting read in normal sample is ≤ 1.
6. Tri allelic frequency in tumor sample is ≤ 0.03.
7. Tri allelic read in tumor sample is ≤ 2.
8. Average Base quality in tumor samples is ≥ 25.
9. Average Base quality in normal samples is ≥ 25.
10. Distance of nearest InDel is > 25.
11. The proportion of soft-clipped reads is ≤ 0.25.
12. Average Mapping quality in both samples is ≥ 15.

We retained the candidate positions for moderate variant allele frequency mutations if they met with the following criteria.

1. The read coverage is ≥ 30
2. The non-reference allele frequency in tumor sample is ≥ 0.07.
3. The normal allele frequency in normal sample is ≤ 0.02
4. Variant supporting read in tumor sample is ≥ 3.
5. Variant supporting read in normal sample is ≤ 1.
6. Tri allelic frequency in tumor sample is ≤ 0.03.
7. Tri allelic read in tumor sample is ≤ 2.
8. Average Base quality in tumor samples is ≥ 25.
9. Average Base quality in normal samples is ≥ 25.
10. Distance of nearest InDel is > 25.
11. The proportion of soft-clipped reads is ≤ 0.25.
12. Average Mapping quality in both samples is ≥ 15.

We should also note that potential mapping errors are excluded by using genomic super duplications, simple repeats, and dbSNP138.

1. genomic super duplications: `http://hgdownload.soe.ucsc.edu/goldenPath/hg19/database/simpleRepeat.txt.gz`
2. simple repeats: `http://hgdownload.soe.ucsc.edu/goldenPath/hg19/database/genomicSuperDups.txt.gz`
3. dbSNP138: `http://hgdownload.soe.ucsc.edu/goldenPath/hg19/database/snp138.txt.gz`

B.3 Filter Conditions for Whole Genome Sequence Data

We retained the candidate positions if they met with the following criteria.

1. The read coverage is ≥ 10
2. The non-reference allele frequency in tumor sample is ≥ 0.05.
3. The normal allele frequency in normal sample is ≤ 0.1
4. Variant supporting read in tumor sample is ≥ 4.
5. Variant supporting read in normal sample is ≤ 1.
6. Average Base quality in tumor samples is ≥ 25.
7. Average Base quality in normal samples is ≥ 25.
8. Tri allelic frequency in tumor sample is ≤ 0.03.
9. Tri allelic read in tumor sample is ≤ 2.
10. Distance of nearest InDel is > 25.
11. The proportion of soft-clipped reads is ≤ 0.25.
12. Average Mapping quality in both samples is ≥ 15.

We should also note that potential mapping errors are excluded by using genomic super duplications, simple repeats, and dbSNP138.

B.4 The Criteria for Calling Somatic Mutation on the Pure Datasets of the TCGA Mutation Calling Benchmark 4 Datasets

1. The read coverage is ≥ 15
2. The non-reference allele frequency in tumor sample is ≥ 0.10.
3. Variant supporting read in tumor sample is ≥ 4.
4. Variant supporting read in normal sample is ≤ 1.
5. Average Base quality in tumor samples is ≥ 25.
6. Average Base quality in normal samples is ≥ 25.
7. Tri allelic frequency in tumor sample is ≤ 0.03.
8. Tri allelic read in tumor sample is ≤ 5.
9. Distance of nearest InDel is > 25.
10. The proportion of soft-clipped reads is ≤ 0.25.
11. Average Mapping quality in both samples is ≥ 15.

B.5 Work Flow of Mutation Calling and Performance Evaluation

In the performance evaluation, we want to evaluate our methods with used filters. Therefore, for the positions with filter labels, we score as very small scores, i. e., 10^{-500}, and then we evaluate all the positions for our method's output. For the used filter label in our performance evaluation, we filtered low_mapping_quality, too_many_softclips_nearby, germline_indel_too_close.

As for the mutation calling for pure sequence data sets, we apply same filters, i.e., low_mapping_quality, too_many_softclips_nearby, germline_indel_too_close.

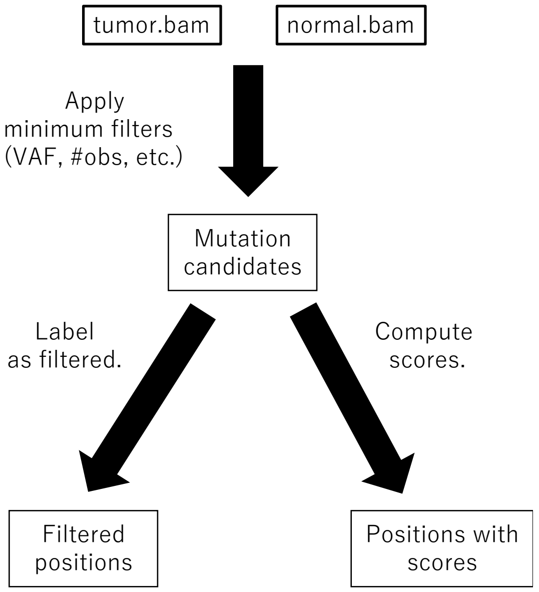

Figure 6:

B.6 Parameters for Alternative Methods in Exome Sequence Data

VarScan2 (v2.3.9): –min-var-freq 0.01 –min-coverage 10 –min-coverage-normal 10 –min-coverage-tumor 10 –somatic-p-value 0.5.

Strelka (v1.0.14): isSkipDepthFilters = 1, and extraStrelkaArguments = –ignore-conflicting-read-names is set on the default setting.

MuTect (v1.1.4): –minimum_mutation_cell_fraction 0.01

OVarCall (v0.1.2): `https://github.com/takumorizo/OHVarfinDer/tree/master/utils/experiments/OVarCall/exome`

B.7 Parameters for Alternative Methods in Whole Genome Data

VarScan2 (v2.3.9): –min-var-freq 0.01 –min-coverage 10 –min-coverage-normal 10 –min-coverage-tumor 10 –somatic-p-value 0.5.

Strelka (v1.0.14): is set on the default setting.

MuTect (v1.1.4): –minimum_mutation_cell_fraction 0.01

OVarCall (v0.1.2): `https://github.com/takumorizo/OHVarfinDer/tree/master/utils/experiments/OVarCall/whole`

Note

The reason for the p-value threshold in VarScan2 is that we want to see all the p-value for all the candidate mutations. (The purpose in this experiment is not collecting only true somatic mutations but plotting the ROC curve.) The reason for different parameter setting in Strelka is that we followed the comments in the Strelka configuration file.

B.8 Basic Information about Original Exome Sequence Data

		F3840			F2816			
Original reads	Mapped reads	PE reads	PE overlap reads	Overlap(%)	PE reads	PE overlap reads	Overlap(%)	Sample
2.08E08	1.98E08	4.83E07	1.99E07	4.11E01	9.89E07	4.23E07	4.28E01	RCC102N
3.09E08	2.91E08	8.96E07	3.48E07	3.89E01	1.46E08	6.06E07	4.16E01	RCC102T
1.33E08	1.28E08	4.13E07	1.86E07	4.50E01	6.40E07	2.97E07	4.63E01	RCC104N
1.30E08	1.25E08	3.98E07	1.73E07	4.35E01	6.24E07	2.81E07	4.50E01	RCC104T
1.51E08	1.44E08	4.29E07	1.75E07	4.08E01	7.22E07	3.03E07	4.19E01	RCC161N
2.04E08	1.93E08	4.78E07	2.07E07	4.32E01	9.63E07	4.29E07	4.46E01	RCC161T
2.78E08	2.65E08	1.06E08	1.35E07	1.28E01	1.32E08	1.74E07	1.32E01	RCC163N
1.50E08	1.45E08	6.29E07	6.41E06	1.02E01	7.27E07	7.51E06	1.03E01	RCC163T
1.55E08	1.50E08	7.21E07	4.50E07	6.25E01	7.51E07	4.69E07	6.25E01	RCC172N
1.52E08	1.48E08	6.95E07	4.15E07	5.97E01	7.38E07	4.41E07	5.98E01	RCC172T
1.56E08	1.49E08	4.57E07	1.85E07	4.04E01	7.47E07	3.10E07	4.15E01	RCC179N
3.01E08	2.83E08	8.95E07	2.99E07	3.34E01	1.42E08	4.96E07	3.51E01	RCC179T
2.37E08	2.26E08	8.99E07	1.10E07	1.23E01	1.13E08	1.43E07	1.27E01	RCC183N
1.95E08	1.90E08	7.01E07	9.31E06	1.33E01	9.48E07	1.30E07	1.37E01	RCC183T
1.74E08	1.66E08	5.59E07	2.11E07	3.77E01	8.32E07	3.24E07	3.90E01	RCC185N
1.73E08	1.67E08	4.72E07	1.54E07	3.26E01	8.35E07	2.87E07	3.44E01	RCC185T
1.68E08	1.62E08	6.52E07	7.00E06	1.07E01	8.08E07	8.90E06	1.10E01	RCC197N
1.42E08	1.37E08	5.92E07	7.06E06	1.19E01	6.84E07	8.32E06	1.22E01	RCC197T
1.71E08	1.66E08	6.51E07	5.16E06	7.93E00	8.30E07	6.74E06	8.12E00	RCC201N
1.21E08	1.17E08	5.05E07	1.71E07	3.38E01	5.83E07	2.00E07	3.43E01	RCC201T
2.25E08	2.13E08	6.84E07	1.33E07	1.94E01	1.07E08	2.04E07	1.92E01	RCC252N
4.12E08	3.83E08	1.00E08	2.44E07	2.43E01	1.92E08	4.44E07	2.32E01	RCC252T
2.96E08	2.79E08	8.78E07	1.81E07	2.06E01	1.40E08	3.02E07	2.16E01	RCC295N
2.92E08	2.75E08	8.13E07	1.94E07	2.39E01	1.38E08	3.24E07	2.35E01	RCC295T
1.48E08	1.40E08	4.27E07	1.87E07	4.39E01	7.01E07	3.17E07	4.53E01	RCC297N
2.42E08	2.28E08	5.14E07	2.21E07	4.30E01	1.14E08	5.10E07	4.48E01	RCC297T
3.73E08	3.48E08	1.02E08	2.05E07	2.02E01	1.74E08	3.72E07	2.14E01	RCC312N
1.91E08	1.81E08	6.30E07	1.28E07	2.03E01	9.07E07	1.81E07	2.00E01	RCC312T
2.40E08	2.24E08	5.91E07	2.56E07	4.33E01	1.12E08	5.10E07	4.55E01	RCC31N
1.76E08	1.67E08	5.92E07	2.37E07	4.01E01	8.33E07	3.46E07	4.15E01	RCC31T
1.90E08	1.83E08	6.88E07	7.65E06	1.11E01	9.14E07	1.05E07	1.15E01	RCC324N
1.97E08	1.89E08	7.60E07	1.59E07	2.10E01	9.45E07	1.96E07	2.08E01	RCC324T
2.16E08	2.08E08	7.91E07	1.42E07	1.79E01	1.04E08	1.77E07	1.70E01	RCC34N
3.80E08	3.61E08	1.16E08	2.55E07	2.20E01	1.80E08	3.78E07	2.09E01	RCC34T
2.26E08	2.16E08	8.69E07	2.87E07	3.30E01	1.08E08	3.63E07	3.36E01	RCC58N
1.23E08	1.19E08	4.40E07	1.21E07	2.76E01	5.93E07	1.67E07	2.83E01	RCC58T
2.68E08	2.54E08	8.01E07	3.04E07	3.79E01	1.27E08	5.01E07	3.95E01	RCC88N
2.39E08	2.26E08	7.59E07	3.14E07	4.13E01	1.13E08	4.84E07	4.27E01	RCC88T
2.29E08	2.17E08	7.21E07	2.74E07	3.80E01	1.09E08	4.29E07	3.95E01	RCC95N
1.33E08	1.27E08	4.06E07	1.69E07	4.16E01	6.37E07	2.74E07	4.30E01	RCC95T

Figure 7: This table shows the basic informations of exome sequence data. The originally downloaded bam is converted to fastq file, with biobambam v0.0.191 with default options. Then extracted sam file is aligned and bam file is made by using bwa(v0.7.8-r455) mem. Original read is the number of reads in fastq files extracted by biobambam. Mapped reads is the number of reads in bam file which satisfy -f(2), -F(256+2048) as for samflag (-f(x) means that a flag of a read contains all flags within x. -F(x) means that a flag of a read does not contain any flag within x.). PE reads is the number of paired-end reads which satisfy -f(1+2+16), -F(3840) (or -F(2816)) as for samflag. PE overlap reads is the number of paired-end reads which satisfy -f(1+2+16), -F(3840) (or -F(2816)) as for samflag and the DNA flagment size is less than the double of the reverse read length.

B.9 Basic Information about Original Whole Genome Sequence Data

		F3840			F2816			
Original reads	Mapped reads	PE reads	PE overlap reads	Overlap(%)	PE reads	PE overlap reads	Overlap(%)	Sample
8.62E08	8.23E08	3.97E08	5.59E07	1.41E01	4.11E08	5.65E07	1.37E01	HCC1143_n100
8.14E08	7.73E08	3.75E08	4.97E07	1.33E01	3.87E08	5.01E07	1.30E01	HCC1143_n95t5
8.20E08	7.55E08	3.67E08	3.36E07	9.17E00	3.77E08	3.39E07	8.98E00	HCC1143_n80t20
8.27E08	7.25E08	3.51E08	1.05E07	2.99E00	3.62E08	1.06E07	2.93E00	HCC1143_n60t40
8.34E08	7.16E08	3.45E08	1.94E06	5.63E-01	3.58E08	2.01E06	5.61E-01	HCC1143_n40t60
8.42E08	7.22E08	3.44E08	7.30E05	2.12E-01	3.61E08	7.77E05	2.15E-01	HCC1143_n20t80
8.47E08	7.32E08	3.44E08	3.40E05	9.87E-02	3.66E08	3.70E05	1.01E-01	HCC1143_n5t95
8.98E08	7.58E08	3.61E08	4.13E05	1.15E-01	3.79E08	4.50E05	1.19E-01	HCC1954_n100
8.46E08	7.15E08	3.42E08	3.97E05	1.16E-01	3.58E08	4.45E05	1.24E-01	HCC1954_n95t5
8.44E08	7.18E08	3.46E08	4.03E05	1.17E-01	3.59E08	4.49E05	1.25E-01	HCC1954_n80t20
8.41E08	7.20E08	3.48E08	3.65E05	1.05E-01	3.60E08	4.05E05	1.13E-01	HCC1954_n60t40
8.38E08	7.23E08	3.48E08	3.40E05	9.77E-02	3.61E08	3.81E05	1.06E-01	HCC1954_n40t60
8.36E08	7.24E08	3.45E08	3.23E05	9.37E-02	3.62E08	3.67E05	1.02E-01	HCC1954_n20t80
8.34E08	7.24E08	3.41E08	3.03E05	8.89E-02	3.62E08	3.45E05	9.53E-02	HCC1954_n5t95

Figure 8: This table shows the basic informations of exome sequence data. The originally downloaded bam is converted to fastq file, with biobambam v0.0.191 with default options. Then extracted sam file is aligned and bam file is made by using bwa(v0.7.8-r455) mem. Original read is the number of reads in fastq files extracted by biobambam. Mapped reads is the number of reads in bam file which satisfy -f(2), -F(256+2048) as for samflag (-f(x) means that a flag of a read contains all flags within x. -F(x) means that a flag of a read does not contain any flag within x.). PE reads is the number of paired-end reads which satisfy -f(1+2+16), -F(3840) (or -F(2816)) as for samflag. PE overlap reads is the number of paired-end reads which satisfy -f(1+2+16), -F(3840) (or -F(2816)) as for samflag and the DNA flagment size is less than the double of the reverse read length.

B.10 ROC Curves in Performance Evaluation

B.10.1 ROC Curves for Simulation Data Sets with 5 % Variant Allele Frequency

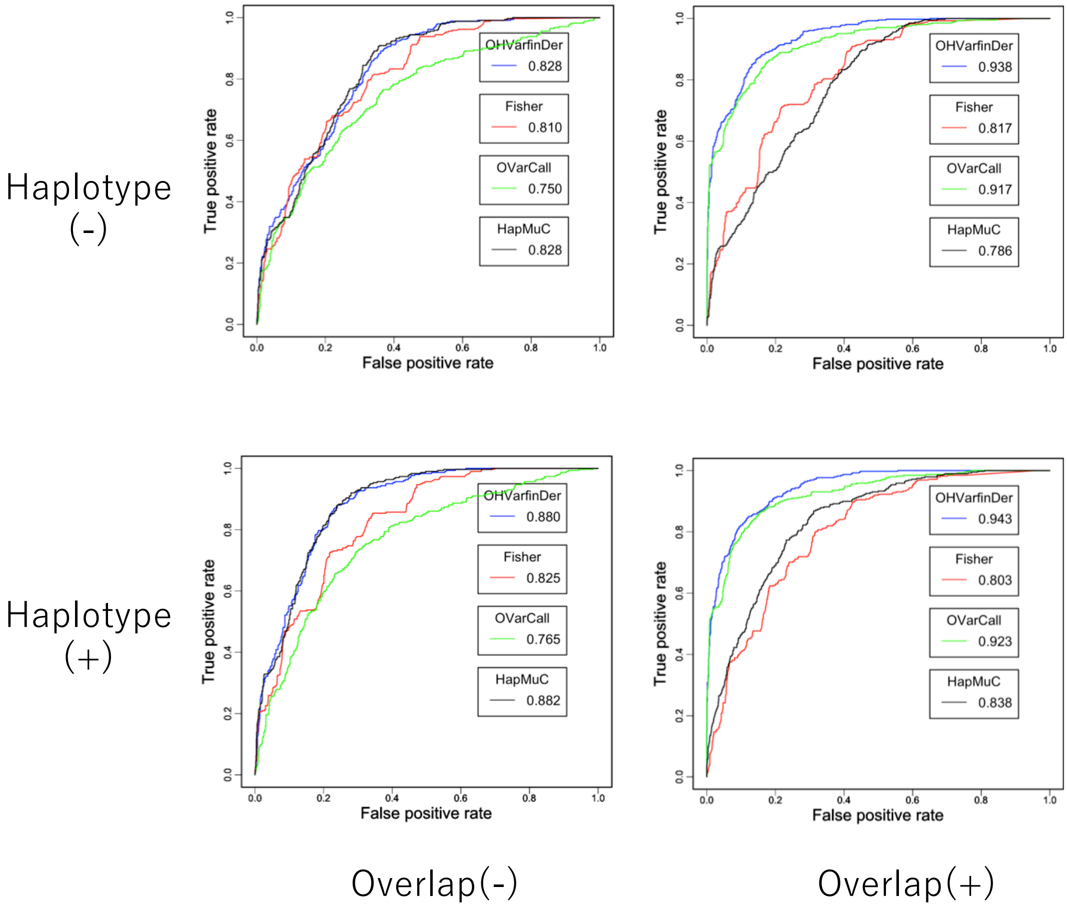

Figure 9:

B.10.2 ROC Curves for Simulation Data Sets with 10 % Variant Allele Frequency

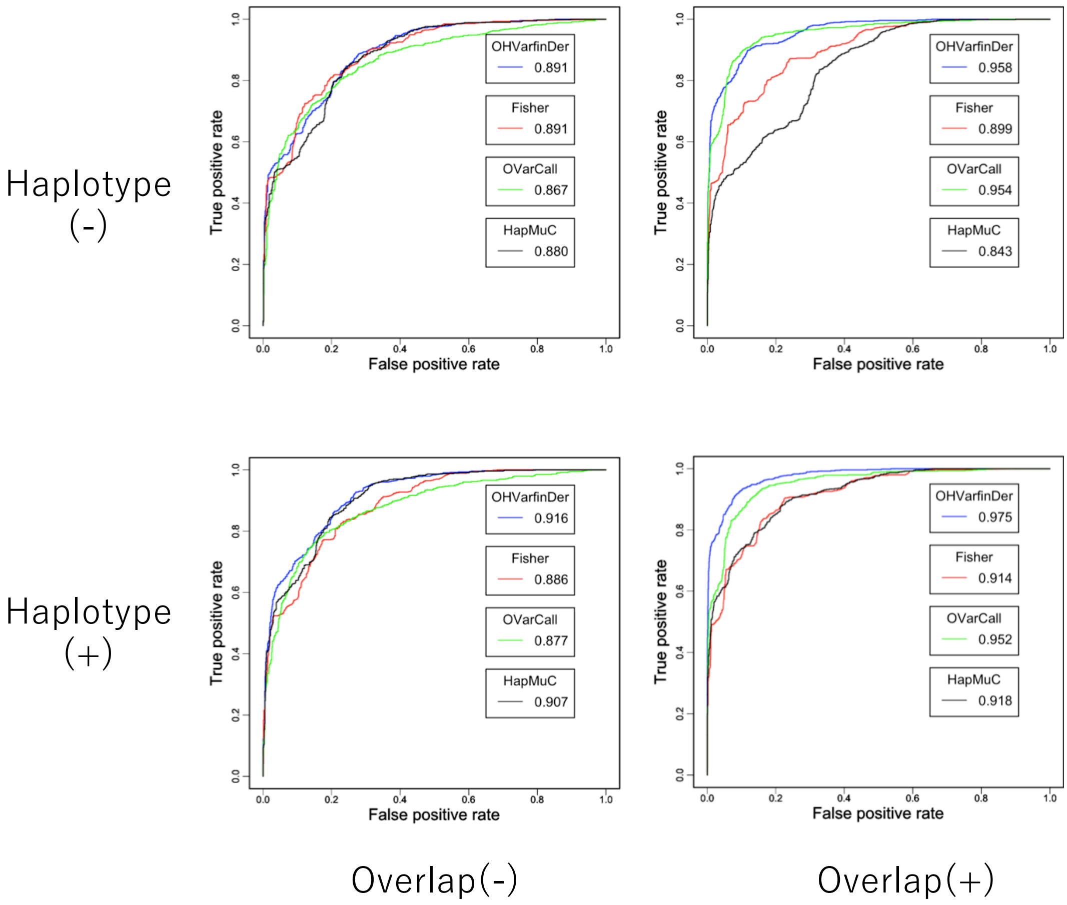

Figure 10:

B.10.3 ROC Curves for Simulation Data Sets with 20 % Variant Allele Frequency

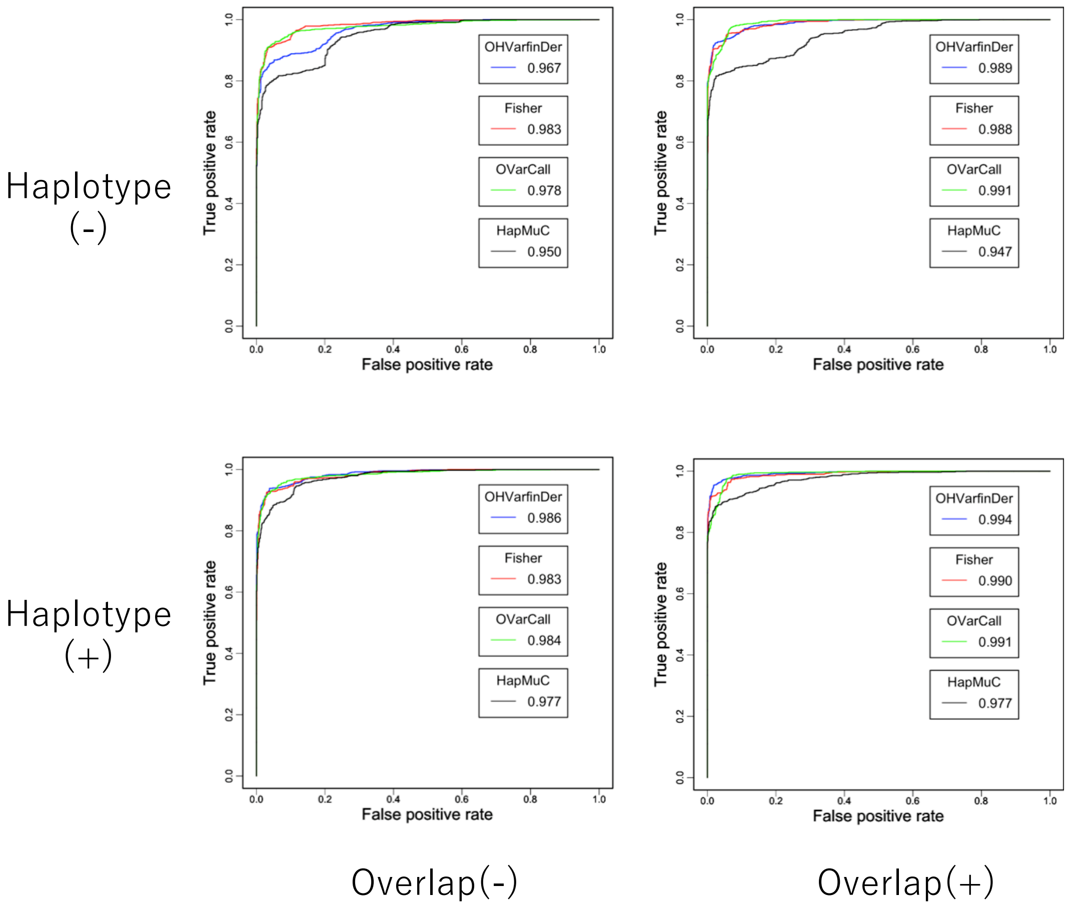

Figure 11:

B.10.4 ROC Curves for SNVs in RCC Data Sets

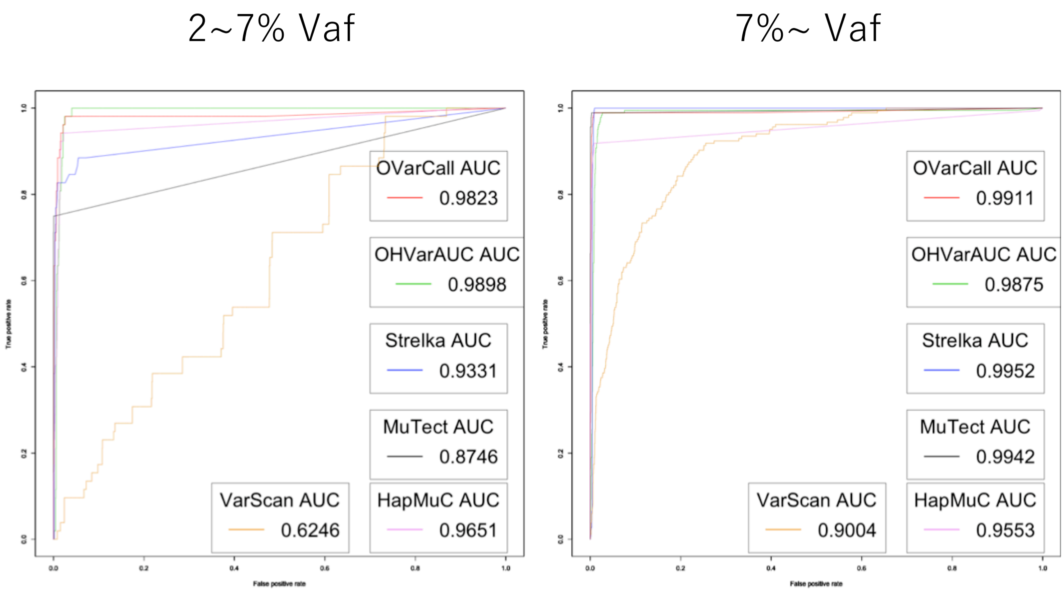

Figure 12:

B.10.5 ROC Curves for SNVs in TCGA Benchmark Data Sets

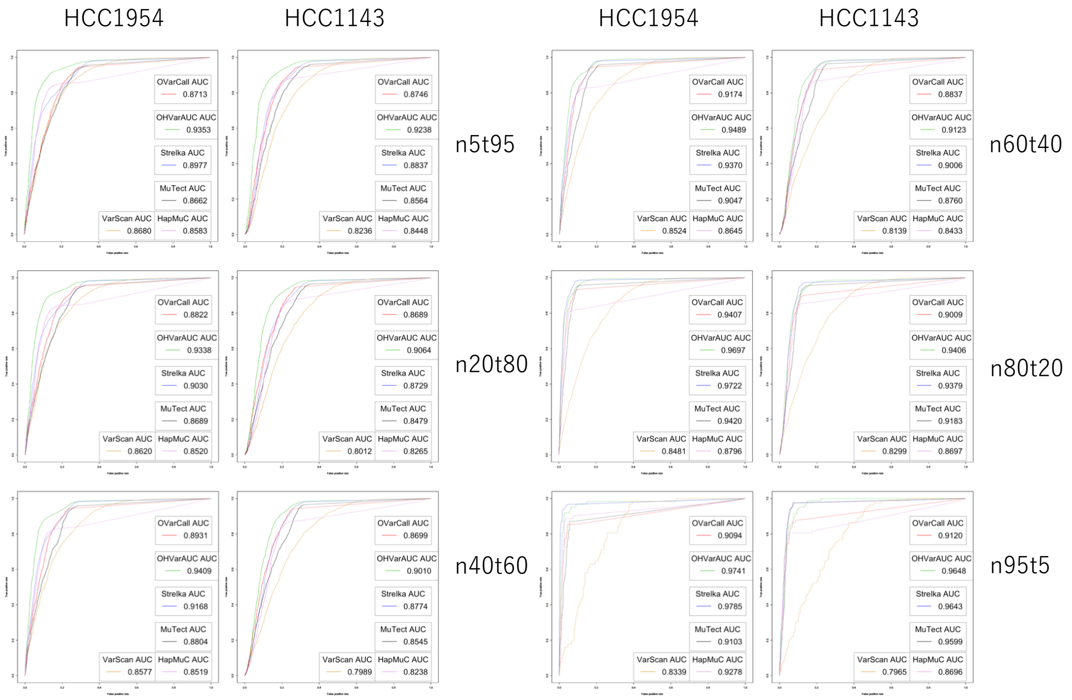

Figure 13:

B.10.6 ROC Curves for InDels in TCGA Benchmark Data Sets

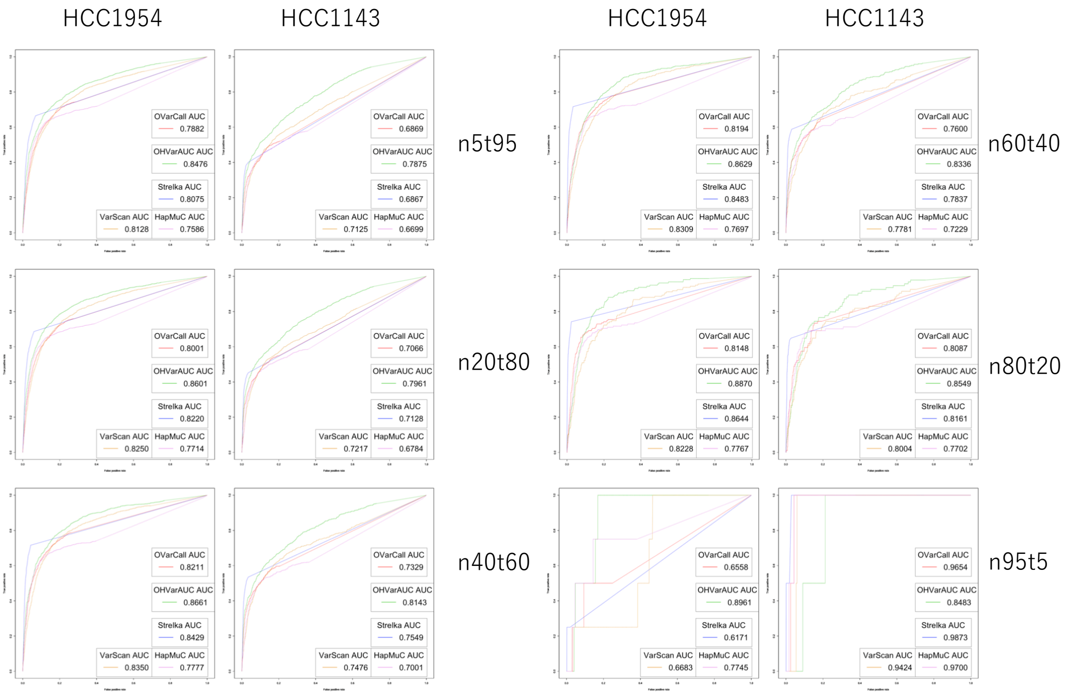

Figure 14:

B.11 Comparison with the Gold Standard Data Sets in ICGC

We compare our method's output with the gold standard [1] in the CLL data set of EGAD00001001858 and other popular mutation callers by drawing Venn-diagram.

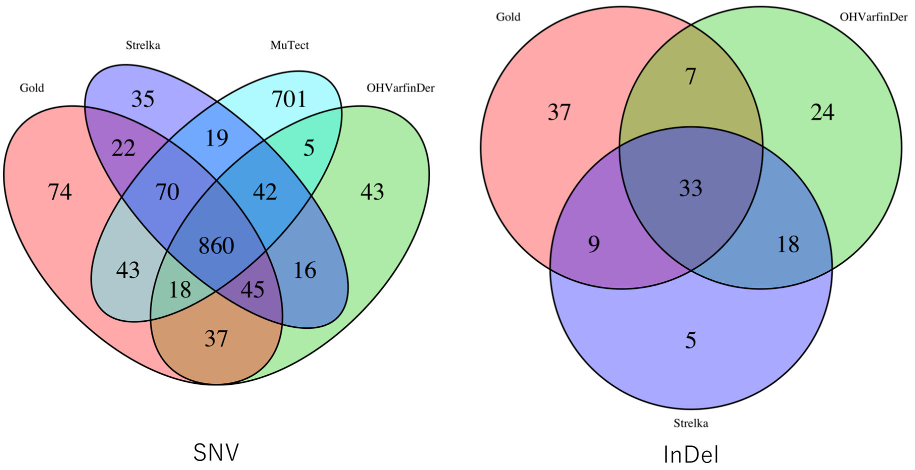

Figure 15: For mutation calling, we used default settings for Stralka and we set –minimum_mutation_cell_fraction as 0.05 for MuTect. Filter condition in OHVarfinDer is same as the criteria in TCGA Mutation Calling Benchmark 4 Datasets. For OHVarfinDer, we selected mutation candidates if Bayes factor ≥ 1. For Strelka, we selected mutation candidates if score in Strelka ≥ 10. For MuTect, we selected mutation candidates if score in MuTect $\geq$ 6.3. We note that genomic super duplications, simple repeats, dbSNP138 are removed.

References

[1] Alioto, T.S., Buchhalter, I., Derdak, S., Hutter, B., Eldridge, M.D., Hovig, E., Heisler, L.E., Beck, T.A., Simpson, J.T., Tonon, L., Sertier, A.-S., Patch, A.-M., Jäger, N., Ginsbach, P., Drews, R., Paramasivam, N., Kabbe, R., Chotewutmontri, S., Diessl, N., Previti, C., Schmidt, S., Brors, B., Feuerbach, L., Heinold, M., Gröbner, S., Korshunov, A., Tarpey, P.S., Butler, A.P., Hinton, J., Jones, D., Menzies, A., Raine, K., Shepherd, R., Stebbings, L., Teague, J.W., Ribeca, P., Giner, F.C., Beltran, S., Raineri, E., Dabad, M., Heath, S.C., Gut, M., Denroche, R.E., Harding, N.J., Yamaguchi, T.N., Fujimoto, A., Nakagawa, H., Quesada, V., Valdés-Mas, R., Nakken, S., Vodák, D., Bower, L., Lynch, A.G., Anderson, C.L., Waddell, N., Pearson, J.V., Grimmond, S.M., Peto, M., Spellman, P., He, M., Kandoth, C., Lee, S., Zhang, J., Létourneau, L., Ma, S., Seth, S., Torrents, D., Xi, L., Wheeler, D.A., López-Otín, C., Campo, E., Campbell, P.J., Boutros, P.C., Puente, X.S., Gerhard, D.S., Pfister, S.M., McPherson, J.D., Hudson, T.J., Schlesner, M., Lichter, P., Eils, R., Jones, D.T.W., Gut, I.G.: A comprehensive assessment of somatic mutation detection in cancer using whole-genome sequencing. Nature Communications **6**(1), 10001 (2015). doi:10.1038/ncomms10001
